# A multiscale computational investigation for protection of carbon steel surface by pyrazolo-pyrimidine derivatives

**DOI:** 10.1038/s41598-025-19022-6

**Published:** 2025-09-17

**Authors:** Mohamed K. Awad, W. S. Abdel Halim, Faten M. Atlam, Mohamed M. Fawzy

**Affiliations:** 1 Chemistry Department, Theoretical Applied Chemistry Unit (TACU), Faculty of Science, Tanta University, Tanta, Egypt; 2 Chemistry Department, Faculty of Science, Zagazig University, Zagazig, Egypt

**Keywords:** Pyrazolo pyrimidine, Corrosion inhibitors, MC, FPMD, LOL, ELF, Chemistry, Materials science

## Abstract

The potential corrosion inhibition properties of pyrazolo-pyrimidine derivatives with extended π system for carbon steel in acidic medium were investigated through a combined DFT, Monte Carlo and molecular dynamic simulations to establish the relationship between electronic properties and adsorption behavior. Geometrical optimization, quantum chemical parameters, (FT-IR) and UV–Vis spectra were explored. The charge transfer and primary sites of adsorption corresponding to enhancement the corrosion inhibition were identified by NBO and Fukui analysis. The Pauli exchange repulsion effect was utilized for the examination of Localized Orbital Locator and Electron Localization Function. To identify bonding and anti-bonding interactions, a study of non-covalent interaction was conducted. Monte Carlo and molecular dynamic simulations predicted strong adsorption energies and explained the stability of inhibitor metal complexes. The ethyl ester substituted inhibitor showed the smallest energy gap (4.640 eV) and the highest negative adsorption energy (− 129.998 kcal·mol^−1^), indicating superior adsorption capability and inhibition efficiency. These findings offer theoretical framework for designing efficient corrosion inhibitors with extended π systems for carbon steel protection.

**Supplementary Information:**

The online version contains supplementary material available at 10.1038/s41598-025-19022-6.

## Introduction

Corrosion is the process by which a metal is destroyed due to chemical or electrochemical reactions with its environment, causing it to lose its original properties and usefulness^1,2^. Corrosion is a natural process that can be extremely costly and dangerous for industries that leads to the degradation of the material’s properties. Researchers have recently focused on the corrosion behavior of iron and steel due to the significant damage it can cause. Carbon steel is susceptible to corrosion due to its high iron content. A large number of effective corrosion inhibitors for steel in acidic solutions are organic compounds. In corrosion science, heterocyclic inhibitors featuring atoms such as N, S, or O, along with π-electron systems and electron-donating groups like CH_3_, NH_2_, and OH that are widely employed in industrial applications to prevent corrosion on to the exposed surface^3,4^. Among of them, pyrazolo pyrimidine derivatives that represent a class of fused heterocyclic in which a five-membered pyrazole ring is condensed with a six-membered pyrimidine in addition to phenyl ring which confer a rich π-electron system. The presence of nitrogen atoms in both rings and additional substituents carboxylic acid in **compound 1**, amide in **compound 2**, and ethyl ester in **compound 3,** are expected to influence electron density distribution, adsorption capability and accordingly its inhibition performance on iron surface^3^. The previous research^5,6^ has examined the corrosion inhibition of organic compounds, attributing to the creation adsorption on the metal surface that is either chemical or physical^7–9^. The inhibitor’s choice is determined by its effectiveness, cost-efficiency, and environmental effect^10^. Pyrazolo pyrimidine is a promising corrosion inhibitors for carbon steel due to its low cost, availability, low toxicity and does not pose any significant health risk in various industrial applications^11^. Further research is needed to fully understand the mechanism of inhibition. It was found that employing quantum chemical study is a potent approach to explore the mechanism of corrosion inhibition^12^. Density functional theory (DFT) and Monte Carlo (MC) simulation model accurately predicts the corrosion inhibition properties of nitrogen-based heterocyclic inhibitors. The molecule with high HOMO energy of Pyridine (− 6.8578), Oxazole (− 6.8572), Pyrimidine (− 6.8300) and pyrazole (− 6.3402) eV value can act good corrosion inhibitors against the corrosion of metal surfaces^13^. In our study, the three pyrazolo pyrimidine derivatives which are namely 3-(3-Methyl-1-phenyl-1H-pyrazolo^3,4^ pyrimidin-4-yloxy)-propionic acid, **compound 1**, propionamide for compound** 2** and ethyl ester derivative of propionic acid, **compound 3 ,** may be highly effective in inhibiting corrosion of carbon steel because they have a positive impact on the mechanical properties of carbon steel^3^. Because of its remarkable stability and densely packed structure, the Fe (110) surface was chosen^14^. First, quantum chemical calculations were used to investigate the relationship between the corrosion inhibition efficiency and the molecular structures of the compounds under study. For the neutral and protonated forms, several global and local reactivity descriptors were computed in the gas and aqueous phases. These descriptors included electron affinity (EA), ionization potential (IP), global softness (σ), global hardness (˕*η*). Additionally, we analyzed local reactivity parameters and dual descriptor of these investigated compounds. MEP, NCI, LOL, ELF and NBO were established created. Monto Carlo simulations were also performed as a part of the study. Following this, FPMD was carried out and the interaction energies were thoroughly assessed and compared. The results obtained are expected to play a significant role in the future investigations of pyrazolo pyrimidine derivatives as anti-corrosive inhibitors.

## Results and discussion

### Molecular geometry of investigated compounds in both protonated and non-protonated form

**Fig. 1:**
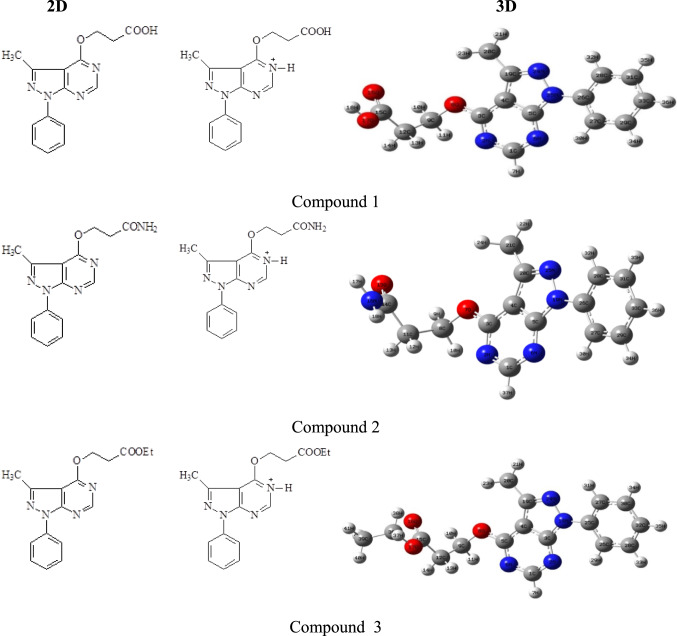
Molecular structures of non-protonated and protonated inhibitors. Generated by Chem Draw Professional 20.0 (PerkinElmer Informatics, Inc.https://informatics.perkinelmer.com. GaussView 5.0 https://gaussian.com

DFT B3LYP/6–311 +  + G(d,p) calculations were used to optimize the inhibitor molecules’ shape in order to produce the best feasible structures. To understanding of how protonation effects on molecular and electronic structures and accordingly on their inhibitory performance, Fig. 1. There are many sites for protonation like N, O, For the titled compounds, nitrogen of pyrimidine ring is highly susceptible for protonation. Also, to see the effect of protonation on molecular structure some selected bond lengths for protonated and non-protonated are tabulated in Table 1. In compounds 1 and 2, the calculations showed an increase in bond length of C1–N2, C1–N6, O8–C9, N25–C26 bonds in protonated form compared to its counterpart in the non-protonated form. This could imply a higher adsorption capability of these groups in protonated form up on adsorption. Compound 3 has relatively long bond length vary from 1.349, 1.323, 1.425, 1.449 Å in non-protonated form to 1.353, 1.327, 1.435, 1.455 Å in protonated form for similar ones. This suggest that such interactions are weakened and that inhibitors attach to metal via nitrogen or oxygen atoms and explain the higher adsorption capability of the investigated inhibitors in protonated form. From above, it is obvious that the protonated inhibitors showed a superior adsorption capability, largely governed by their ability to interact through nitrogen and oxygen atoms with Fe atoms on the carbon steel surface, aided by both the nature of the substituents and physisorption interactions.

**Table 1:** Some selected bond lengths for investigated compounds obtained from DFT/6-311G +  + (p,d) in non-protonated and protonated form in gas phase.

Bond length(Å)	Compound 1		Compound 2		Compound 3	
	Gas	Protonated	Gas	Protonated	Gas	protonated
C(1)–N(2)	1.35	1.353	1.349	1.353	1.323	1.327
C(1)–N(6)	1.323	1.327	1.323	1.327	1.349	1.352
N(2)–C(3)	1.325	1.319	1.327	1.319	1.344	1.334
C(3)–O(7)	–	–	1.337	1.317	–	–
C(3)–O(8)	1.340	1.314	–	–	–	–
C(3)–N(18)	–	–	–	–	1.370	1.362
C(5)–N(6)	1.344	1.334	1.345	1.334	1.326	1.320
C(5)–O(8)	–	–	–	–	1.338	1.312
C(5)–N(19)	–	–	–	–	–	–
C(5)–N(25)	1.369	1.362	1.369	1.362	–	–
O(7)–C(8)	–	–	1.449	1.455	–	–
O(8)–C(9)	1.447	1.461			1.447	1.466
C(14)–O(15)	–	–	1.218	1.224	–	–
C(14)–N(16)	–	–	1.364	1.357	–	–
C(15)–O(16)	1.205	1.206	–	–	1.207	1.209
C(15)–O(17)	1.355	1.347	–	–	1.347	1.337
O(17)–C(36)	–	–	–	–	1.454	1.461
C(19)–N(24)	1.316	1.341	–	–	1.315	1.341
N(18)–N(24)	–	–	–	–	1.377	1.375
N(24)–N(25)	1.377	1.374	–	–	–	–
N(25)–C(26)	1.425	1.436	–	–	–	–
N(18)–C(25)	–	–	–	–	1.425	1.436
N(19)–N(25)	–	–	1.377	1.376	–	–
N(19)–C(26)	–	–	1.425	1.435	–	–
C(20)–N(25)	–	–	1.316	1.339	–	–

Bond length variations among the inhibitors are influenced by the electronic nature of the substituents. The introduction of substituents OH (compound 1), NH_2_ (compound 2), and OC_2_H_5_ (compound 3) significantly affected the structural and adsorption characteristics, with electron-donating groups (EDGs) generally increasing electron density at the adsorption centers. In the gas and protonated phase of inhibitor 1, the C15–O17 bond lengths equal 1.355 Å and 1.347 Å respectively, and 1.347 and 1.337 respectively, for inhibitor 3, which are shorter than C14–N16 bond length in inhibitor 2. This can be attributed to the lower electronegativity of nitrogen compared to oxygen, which reduces bond polarity and leads to a slightly longer C–N linkage. In all cases, the bond lengths were longer in the neutral (gas-phase) forms than those in the protonated species, indicating a looser electronic environment around the donor atoms in the absence of protonation. Upon protonation expected under acidic conditions, the increased electrostatic attraction and redistribution of electron density resulted in shorter bonds, reflecting a more compact geometry at the adsorption centers. Notably, the ethoxy-substituted derivative in compound 3 exhibited the shortest protonated C–O bond length among all derivatives, suggesting that the inductive electron-donating effect of the OC_2_H_5_ group sustains electron density at the donor site even after protonation, thereby favoring strong adsorption. From a substituent-effect view, the electron-donating OC_2_H_5_and OH groups enhance nucleophilicity and facilitate metal ligand interactions more effectively than NH_2_ in this system, despite NH_2_ also being classified as an EDG. This difference arises from the combined electronegativity and resonance effects, where oxygen-based EDGs provide better overlap and stronger donor capacity toward the Fe surface. Moreover, the incorporation of these substituents also increased the adjacent carbonyl (C=O) bond length in the protonated species provides more binding sites, Table 1, which explains a higher adsorption capabilities of protonated inhibitors than non protonated ones.

### Frontier molecular orbitals (FMOs)

**Fig. 2:**
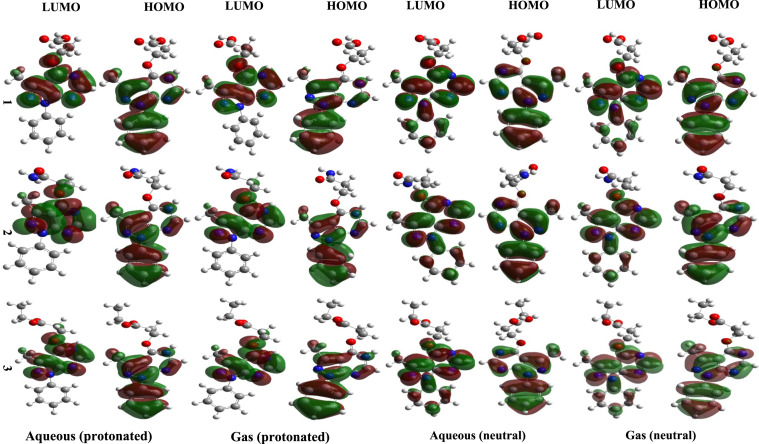
HOMO and LUMO of neutral and protonated form of investigated compounds in gas and aqueous phase. Generated using GaussView 5.0 (Gaussian, Inc., Wallingford, CT, USA; https://gaussian.com/

Frontier molecular orbitals, namely the highest occupied molecular orbital (HOMO) and, the lowest unoccupied molecular orbital (LUMO) play a crucial role in studying the nature of interactions. Figure 2 depicts the localization of electron density changes in the HOMO and LUMO in gas and aqous phase for the neutral and protonated investigated compounds indicating the most relevant sites for adsorption. Calculations have revealed that in case of HOMO, the charge density distribution is localized on pyrazolo pyrimidine heterocyclic moiety, in addition to benzene ring and oxygen atom in pyrimidine ring. This localization suggests that this specific region of these molecules could act as a nucleophile, meaning it has the potential sites to donate electrons in a chemical reaction providing the best opportunity for donation through its N atoms and π electrons systems to the vacant d-orbitals of Fe atoms on the carbon steel surface. Regarding the LUMO, significant distribution showed that the reactive sites are concentrated on the entire hetero-cycle pyrazolo pyrimidine and phenyl ring for three inhibitors in neutral form. While in case of protonated form phenyl ring doesn’t participate in the distributions of LUMO densities. π-back donation from Fe to inhibitor is possible, particular into low-lying LUMO orbital localized on the π system of the pyrazolo-pyrimidine core and the carbonyl group. The fused pyrazolo-pyrimidine heterocycle, containg multiple electronegative atoms, exhibits an electron withdrawing character. The electron withdrawing nature of substituents COOH Compound 1, CONH_2_ Compound 2 and COOEt compound 3, stabilizes the LUMO level, making them suitable for accepting electron density from the filled d-orbital of Fe atom via back donation. According to Al-Qurashi et.al^15^, higher electrophilicity index (ω) and lower electron donating capability (e^−^) values favor back donation from the metal to the inhibitor, whereas lower (ω) and higher (e^−^) values indicate a greater tendency for the inhibitor to donate electrons to the metal. It is concluded from Fig. 2, that the phenyl pyrazolo pyrimidine moiety could act as potential active site adsorbed to the iron metal on carbon steel.

E_HOMO_ and E_LUMO_ energies, and the LUMO–HOMO energy gap (∆Egap), were calculated using FMOs theory and are recorded in Table 2. Based on the theory, molecules with higher (less negative) E_HOMO_ values have shown greater potential to provide electrons to acceptor one. As shown in Table 2, E_HOMO_ increase with the replacing of OH group compound 1, and NH_2_ compound 2 by ethoxy, (OEt) compound 3 in both the gas and aqueous phases for protonated and non-protonated inhibitors. A molecule’s reactivity can also be predicted using the energy gap (∆E). In general, a smaller ∆E suggests greater chemical reactivity and, consequently, enhanced corrosion inhibition efficiency. Therefore, compound 3 was observed to be the most effective inhibitor, exhibiting the least ΔE values, 4.64, and4.713 eV, respectively, in gas and aqous gas phase for non-protonated while for gas and aqous protonated are 3.927 and 4.623 eV, respectively.

**Table 2:** E_HOMO_ and E_LUMO_ as well as ΔE _gap_ of neutral and protonated form of investigated compounds.

Compound	E_HOMO ,_ (eV)	E_LUMO_ (eV)	ΔE (eV)
Gas (neutral)			
1	− 6. 261	− 1.611	4.651
2	− 6.262	− 1.615	4.647
3	− 6.214	− 1.566	4.64
Gas (protonated)			
1	− 9.632	− 5.738	3.894
2	− 9.562	− 5.630	3.932
3	− 9.571	− 5.643	3.927
Aqueous (neutral)			
1	− 6.427	− 1.711	4.717
2	− 6.416	− 1.700	4.715
3	− 6.413	− 1.710	4.713
Aqueous (protonated)			
1	− 7.090	− 2.466	4.624
2	− 7.068	− 2.432	4.636
3	− 7.078	− 2.455	4.623

### Global reactivity descriptors

Using Koopmans’ theorem^16^, values for ionization potential (IP), electron affinity (EA), hardness(η), and softness (σ) were computed. Softness is recognized as a key parameter in evaluating the reactivity of chemical systems. It has been noted that systems with higher softness values tend to be more reactive, while lower softness values indicate decreased reactivity. As shown in Table 3, a general trend was observed where softness values increase as:1 < 2 < 3 in both in gas and aqueous phases for neutral and protonated investigated inhibitors. For example, the softness values for the neutral compounds 1,2 and 3 were 0.430, 0.430 and 0.431 eV, respectively in gas phase, While in aqueous phase, they are 0.423,0.424 and 0.425 eV ,respectively. The reactivity of chemical system is also, accompanied with ionization potential. Higher ionization potential values tend to be less reactive, while lower IP values indicate increased reactivity. In gas phase, the trend of decreasing IP: 1 > 2 > 3. Here, we can conclude that when IP is low means the molecules easier to donate electrons to carbon steel. That’s why it complementary with higher softness. On the other hand, the lower the electronegativity index of a molecule, the stronger its tendency to donate electrons. It is essential to remember that effective corrosion inhibitors should have a high electron-donating power characters^17^. The calculations showed that compound 3 has a significantly low electronegativity in both gas and aqoues phase. This is likely a significant factor contributing to the improved its inhibition efficiency. According to Lukovits^18^, If ΔN the number of electron transfer is less than3.6e^−^, inhibition efficiency increases with higher electron donating ability at the metal surface^19^. The calculations showed that all investigated molecules has ΔN less than3.6 e^−^, Table 3, which expect to inhibit iron corrosion through electron donation to the electron acceptor iron surface. The calculated total negative charge (TNC)which is defined as sum of negative charges present in the whole molecule, proved that compound 3 has large TNC values, − 4.309 e^−^ and − 4.69 e^−^ ,in gas and water solvent, respectively, which indicates the presence of active center and accordingly, a high adsorption capability. This behavior can be directly related to the nature of its substituent group; COOEt, compound 3 compared with COOH (compound 1) and CONH_2_ (compound 2). The ethoxy ester group exhibits a partial electron-donating inductive effect (+ I) via the alkyl chain, which increases the electron density over the molecular framework, particularly at potential adsorption sites. This effect enhances the localization of negative charge and thus increases the TNC, indicating more active centers available for interaction with the Fe surface. In contrast, the COOH and CONH_2_ groups are more polar and prone to hydrogen bonding, protonation, or strong solvation in aqueous acidic media, which can draw electron density away from the molecular framework, thereby lowering the TNC and reducing the overall electron-donating ability. Consequently, the substitution with COOEt not only increases the TNC but also improves the molecule’s adsorption potential. Consequently, it can be inferred that compound 3 can adsorb onto the steel surface through multiple active sites, which will be discussed later resulting in significant surface coverage and improved protection. In certain cases, a molecule may accept a charge at one site and subsequently return that charge through the same or a different site. Gómez et al.^20^ proposed a simple expression to estimate charge back-donation ΔE _Back-donation_ = (− η / 4). For the process to be energetically favorable, η (equal to ΔE/2) must be greater than zero, and ΔE_Back-donation_ should be negative. The values of η and ΔE_Back-donation_ shown in Table 3 indicate that this condition is achieved. Among the investigated inhibitors, compound 3 was identified as the most effective inhibitor with the value of ΔE_Backdonation_ = − 0.58 eV and − 0.579 eV, respectively, in gas and aqueous phase for neutral inhibitor, while ΔE_Back-donation_ = − 0.491 eV and − 0.590 eV,respectively, in gas and aqueous phase, respectively, for protonated ones. It is concluded from the above discussion that the insertion of OC_2_H_5_ substituent, compound 3, generates a partial electron-donating, and increases the electron density effect through the oxygen atom, leading to an elevation in the HOMO energy level and a reduction in ionization potential and the energy gap (ΔE) in gas and aqueous phase for protonated and non-protonated. This in turn enhances the molecule’s global softness while decreasing its global hardness. Moreover, the substituent contributes to a higher (TNC) and ΔE _Back-donation_ which enhances the chance of inhibitor adsorption on Fe surface.

**Table 3:** The calculated quantum chemical parameters obtained from DFT/6-311G +  + (p,d)for neutral and protonated compounds in gas and water solvent.

Compound	**I.P**	**EA**	η	**σ**	**X**	**µ**	$\mathcal{W}$	**∆N**	**TNC**	**ΔE**_**back donation**_	D
Gas(Neutral)											
1	6.261	1.611	2.325	0.430	3.936	− 3.936	3.332	0.244	3.028	− 0.582	1.977
2	6.262	1.615	2.323	0.430	3.939	− 3.939	3.338	0.243	3.73	− 0.581	2.870
3	6.214	1.570	2.322	0.431	3.890	− 3.890	3.255	0.254	4.309	− 0.580	2.580
Aqueous(Neutral)											
1	6.427	1.712	2.359	0.423	4.070	− 4.070	3.510	0.212	3.450	− 0.590	2.730
2	6.416	1.700	2.357	0.424	4.058	− 4.058	3.490	0.215	4.040	− 0.589	3.960
3	6.413	1.710	2.356	0.425	4.060	− 4.056	3.500	0.214	4.690	− 0.580	3.220
Gas(protonated)											
1	9.632	5.738	1.947	0.514	4.921	− 4.921	6.220	0.038	3.66	− 0.487	7.163
2	9.562	5.63	1.966	0.508	4.884	− 4.884	6.068	0.047	4.194	− 0.492	9.227
3	9.571	5.643	1.964	0.509	4.889	− 4.889	6.086	0.046	4.281	− 0.491	6.969
Aqueous (Protonated)											
1	7.09	2.466	2.312	0.432	4.770	− 4.770	4.936	0.066	3.984	− 1.156	11.529
2	7.069	2.432	2.318	0.431	4.750	− 4.750	4.867	0.069	4.226	− 0.159	13.309
3	7.078	2.455	2.311	0.433	4.760	− 4.760	4.914	0.063	4.661	− 1.155	10.308

### Molecular electrostatic potential

**Fig. 3:**
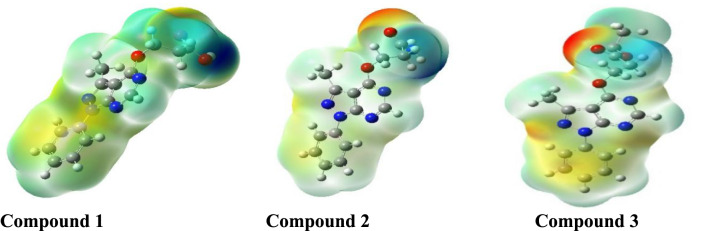
The molecular electrostatic potential of the investigated neutral compounds in gas phase. Generated using GaussView 5.0 (Gaussian, Inc.,Wallingford, CT, USA; https://gaussian.com/

The molecular electrostatic potential mapping is a 3D visual tool used to analyze the electrostatic properties of a molecule. It helps to differentiate the net electrostatic impact across a molecule from the total charge distribution. In this context, “electrostatic” refers to the distribution of electric charges within a molecule which plays a powerful role in determining its reactivity^21^. In Fig. 3, the red and yellow colors represent regions of the molecule with the highest electron density, indicating a nucleophilic reaction tendency. On the other hand, the blue color represents regions with the most positive electrostatic potential, indicating an electrophilic reaction tendency, while the neutrally potential are shown by the green lines. A protective layer forms as a result of electron exchange between the investigated inhibitors and the steel surface during chemical adsorption. From Fig. 3, the most electron-rich regions are mainly localized around the oxygen atoms of –C=O, OH and C=N groups, conjugate and heteroatoms bonds. These types of bonds are particularly prevalent in such areas due to the abundance of electrons. In other words, the hydrogen atoms and some carbon atoms have a positive electrostatic potential and are likely to be targeted by nucleophiles, which agrees well with FMOs discussion.

### Analysis on LOL, ELF

The Localized Orbital Locator, LOL, is a widely used topological approach for exploring and visualizing covalent interactions in molecules^22^. The LOL equation is given as the following

$${\text{LOL = }}\frac{{\tau_{r} }}{{1 + \tau_{r} }}$$

where $\tau_{r}$ represent local kinetic energy.

**Fig. 4:**
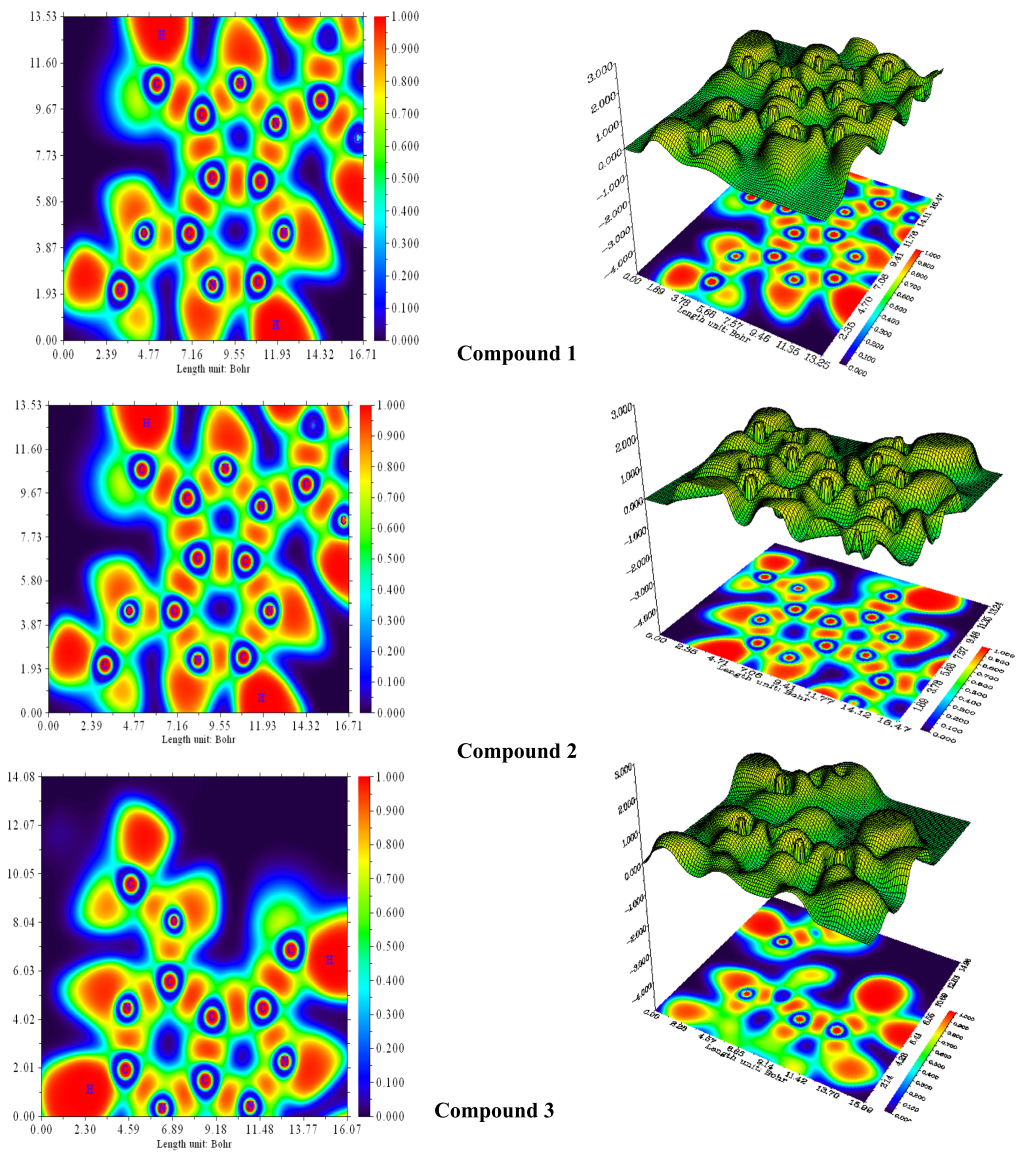
Three-dimensional color-filled surface and shaded projection maps of (ELF) and (LOL) for the investigated compounds**.** Generated using Multiwfn 3.6 (Tian Lu, Beijing Kein Research Center for Natural Sciences; http://sobereva.com/multiwfn.

Visualization of LOL and Electron Localization Function (ELF) on a molecular plane defined by three key atoms located at the predicted adsorption site. The LOL distribution is presented as a surface-shaded projection, while the ELF is depicted using a color-filled contour map to enhance the electron localization in Fig. 4. The plots were generated using *Multiwfn 3.6* (http://sobereva.com/multiwfn)^23^.

LOL and ELF maps exhibit similar electron density distributions. The color gradient from blue to red illustrates the values indicating bonding and non-bonding electrons, with high ELF values shown in red and low values in blue color^24,25^. High localization values (red regions) are concentrated around heteroatoms (O, N) and hydrogen atoms bonded to them, indicating the presence of covalent bonds and lone electron pairs. Moderate localization (orange) is observed in C–N and C–O bonds, while low localization (blue) is found over aromatic carbons (phenyl, pyrimidine, and pyrazole rings) due to delocalized π-electrons. These patterns suggest that adsorption onto the Fe surface is primarily driven by the lone pairs on O and N atoms, complemented by π–π stacking interactions. The spatial orientation of these electron-rich centers facilitates strong coordination with the surface, Additionally, LOL calculation is agreed well with FMO and NBO natural charge which will discussed later.

### Reduced density gradient

The reduced gradient of electron density (RDG) function is a topological measure that used to examine and highlights non-covalent interactions (NCI) like steric hindrance, hydrogen bonds and van der Waals forces^26^. Being a dimensionless quantity, the RDG is expressed in terms of the electron density ρ(r) and its gradient ∇ρ(r) as shown below:

$$RDG_{{\left( r \right)}} = \frac{1}{{2\left( {3\pi r^{2} } \right)^{{\frac{1}{3}}} }}\frac{{\left| {\nabla \rho _{{\left( r \right)}} } \right|}}{{\rho _{{\left( r \right)}}^{{{\raise0.7ex\hbox{$4$} \!\mathord{\left/ {\vphantom {4 3}}\right.\kern-\nulldelimiterspace} \!\lower0.7ex\hbox{$3$}}}} }}.$$

**Fig. 5:**
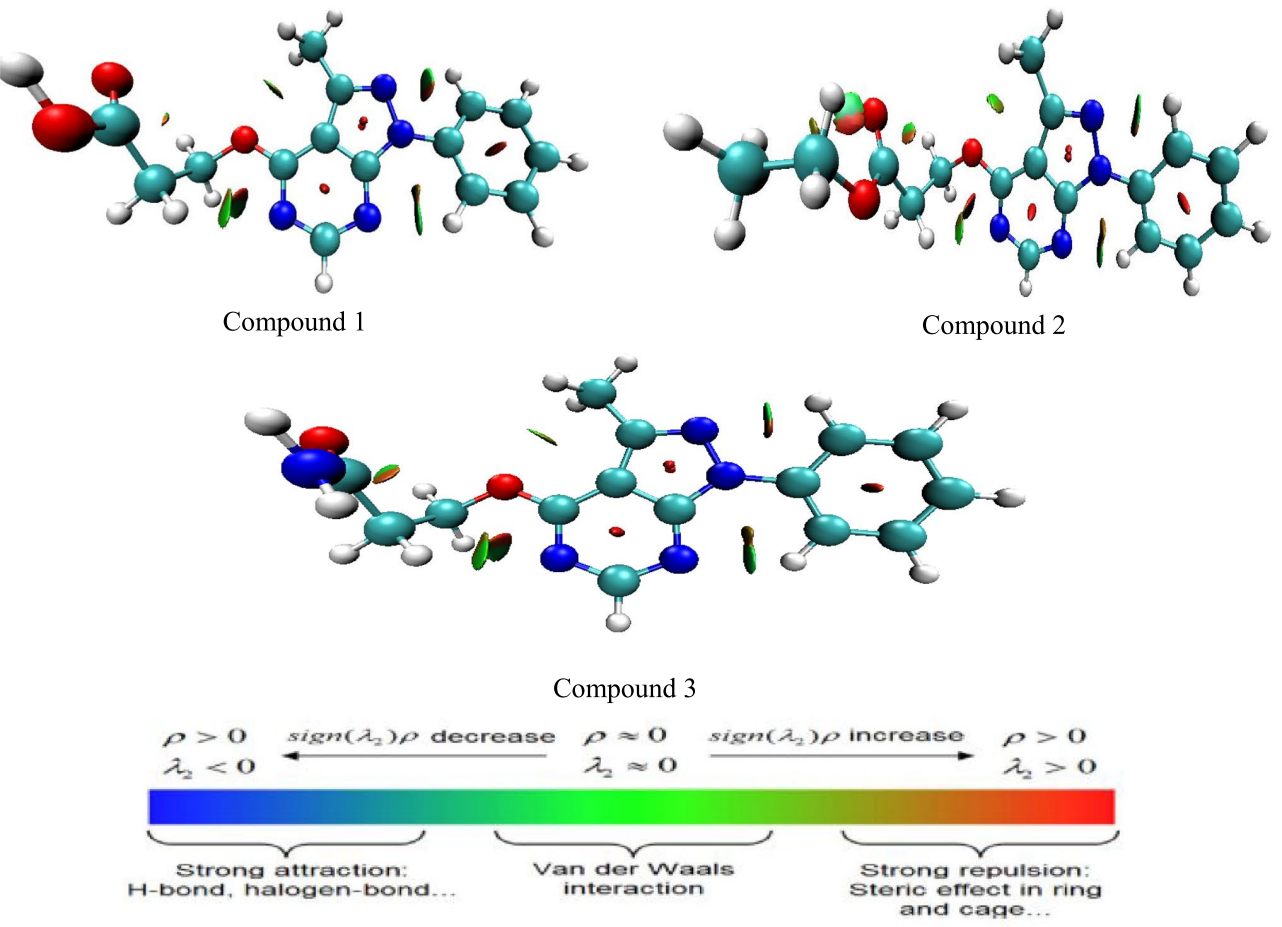
Reduced density gradient (RDG) of the investigated compounds. Generated using Multiwfn 3.6 http://sobereva.com/multiwfn with visualization performed in VMD 1.9.3 (University of Illinois at Urbana–Champaign; https://www.ks.uiuc.edu/Research/vmd/

Figure 5 is a representation of covalent and non-covalent interactions (NCI) ,where NCI isosurface was computed using *Multiwfn 3.6* (http://sobereva.com/multiwfn software[23]in combination with Visual Molecular Dynamics (VMD) software^27^. Using the electron density and the nature (sign) of the second eigenvalue (λ2) obtained from the Hessian matrix^28^, where λ1 is the greatest eigenvalue of the Hessian matrix, the RDG function is computed. Different kinds of non-covalent interactions can be categorized by different signs of λ_2_. Additionally, if λ2 > 0, the interaction may be attractive; if λ2 < 0, it may be repulsive. The predicted RDG plots for the investigated molecules are illustrated in Fig. 5, where the red color (steric effect) on both phenyl rings and the pyrazolo pyrimidine moiety indicates repulsion between the π-electron clouds. In addition, the green areas show van der Waals interactions between the phenyl and pyrazole rings.

The NCI analysis supports that all three derivatives have multiple non-covalent interaction domains and donor sites Fig. 5. These van der Waals interactions, as shown above suggest that the molecules can achieve favorable orientations on the Fe(110) surface, maximizing surface coverage physisorption. Furthermore, the extended conjugation π-electron system and multiple nitrogen atoms in the heterocyclic serve as electron-rich sites facilitating π-metal, and chemisorpative interactions with Fe atom. Variations in terminal functional groups further influence adsorption capability: the COOH group in compound 1 may form strong coordination, the CONH_2_ group in compound 2 provides dual O/N donor sites for interaction with Fe atoms, and the ester group in compound 3 retains coordination ability through its carbonyl oxygen. These combined electronic and NCI features contribute to the high adsorption propensity and effective corrosion inhibitor (Fig. 5).

### Fuki function

**Fig. 6:**
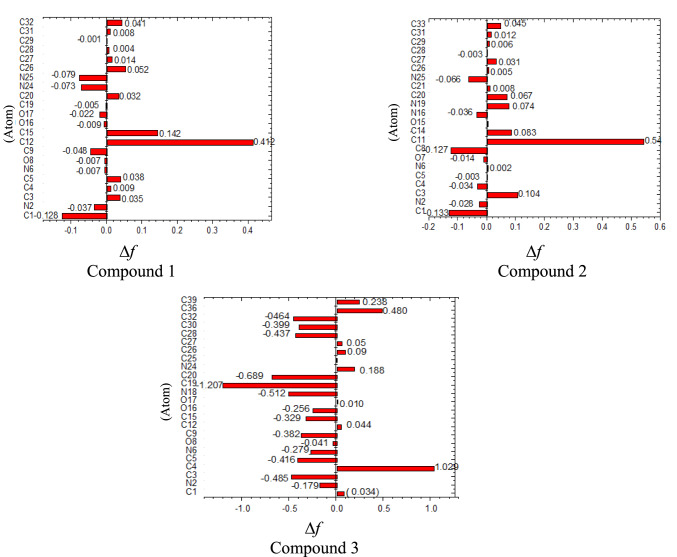
The calculated dual descriptor ∆f for the investigated compounds. Generated by https://getintopc.com/softwares/3d-cad/originpro-2021-free-download/

The Fuki function(r),describes density electron shift due to an infinitesimal variation in the number of electrons^29^.

$${\text{F}}\left( {\text{r}} \right) = \left( {\frac{{\partial {\uprho }\left( {\text{r}} \right)}}{{\partial {\text{N}}}}} \right)_{{{\text{vr}}}}$$

where ρ(r) is the electron density function, N = ∫ρ(r)dr indicates the total number of electrons, and r defines the spatial variable under the influence of the external potential^30^. The reactivity of electron-rich centers and electron-deficient centers (tend to electrophilic attack and nucleophilic attack) respectively, can be assessed by applying the Fukui function and the following formulas^31^.

$$\begin{gathered} \begin{array}{*{20}c} {f^{ + } = {\text{q}}\left( {{\text{N}} + {1}} \right){-}{\text{qN}}} & {\text{for nucleophilic attack}} \\ \end{array} \hfill \\ \begin{array}{*{20}c} {f^{ - } = {\text{qN}} - {\text{q}}\left( {{\text{N}} - {1}} \right)} & {\text{for electrophilic attack}} \\ \end{array} \hfill \\ \end{gathered}$$

where the atomic charges of the (anionic, neutral, and cationic) molecules are donated by q(N + 1), q(N), and q(N − 1), respectively (evaluated from Mulliken population analysis). When a molecule gains electrons, they are most likely to localize in regions where (f⁺) is high. In chemical density functional theory (DFT), Fukui functions serve as essential indicators of regioselectivity in reactions governed by electron transfer. Adual descriptor, Δf(r), was recently proposed by Morell et al.^32^. This descriptor is expressed as Δf(r) = f⁺ − f⁻, where Δf(r) is greater than 0), the site are considered electrophile (favored for nucleophile attack Meanwhile, those with Δf < 0 are nucleophiles (favored for electrophilic attack). It is shown from data that most preferred site for nucleophile in compound 3 are C_1,_ C_4_,C_12,_ C_26,_ C_27,_ C_36_ (positive value ∆* f* (r) > 0). Conversely, the calculations showed that electrophilic sites attack in compound 3 are N_2_, C_3_, C_5_, N_6,_ O_8,_C_9,_ O_16_, O_17_ atoms (negative value ∆*f* (r) < 0). Furthermore, the electrophilic and nucleophilic sites attack for compound 1and 2 are shown in Fig. 6 and Tables S1, S2 and S3.

The Fukui function analysis indicates that substituent type markedly influences the distribution of electron density and the location of the most reactive sites. The substitutents COOH group in compound 1, or the CONH_2_ group in compound 2, with the COOEt group in compound 3 results in a clear increase in electron density at the oxygen atoms. For example, the ∆f(r) value for O_16_ in compound 3 is − 0.026, compared with − 0.009 in compound 1 and 0.002 for O14 in compound 2. Additionally, the ethoxy group in compound 3 generates extra reactive centers, increasing the electrophilic character of C_36_ and C_39_, with ∆f(r) values of 0.480 and 0.238, respectively. Compound 3 its greater electron-donating ability , particularly through the carbonyl oxygen and the π-system of the pyrazolo-pyrimidine scaffold, is highly favorable for π–metal interactions and binding with Fe(110) surface on carbon steel . Based on the Fukui function data, compound 3 is expected to exhibit the highest inhibition efficiency. The enhanced performance arises from the accumulation of reactive centers which facilitates electron transferring or accepting contributing to stronger adsorption. These results agree with FMO and MEP investigations. This is verified by the calculations of the local softness which defined as the multiplication of the Fukui’s role by the global softness (σ), can be formulated as

$${\text{S}}^{ + } = (f^{ + } ){{\varvec{\upsigma}}}\quad {\text{S}}^{ - } = (f^{ - } ){{\varvec{\upsigma}}}\quad \Delta {\text{S}} = \left( {{\text{S}}^{ + } } \right){-}\left( {{\text{S}}^{ - } } \right) = \Delta f{{\varvec{\upsigma}}}$$

High values of s^+^ and s^−^indicate a high nucleophilicity and electrophilicity, respectively . According to the dual descriptor (∆S), (positive value (∆S) > 0), the site is likely to undergo nucleophilic attack, whereas if(negative value (∆S) < 0 then the site tends to attract electrophilic attack. The interaction between inhibitor and metal surface is more easily via soft atoms. The trend of softness agrees well with dual descriptors Tables S1, S2 and S3.

### Natural bond orbital (NBO)

NBO provides an effective approach for examining both intra- and intermolecular bonding by offering a precise depiction of electron density in the form of “natural Lewis structure”. Donor–acceptor interactions within the NBO basis are evaluated through the second-order Fock matrix^33^. Moreover, atomic charges derived from NBO analysis significantly influence chemical reactivity^34^. This approach aids in understanding how filled orbitals of one subsystem interact with vacant orbitals of another, reflecting intermolecular delocalization or hyperconjugation. These interactions cause electron density to shift from localized Lewis orbitals to empty non-Lewis orbitals. The stabilization energy (E2) for each donor (i) and acceptor (j) pair is computed using the following equation:

$${{{\text{E}}_{{2}} = {\text{q}}_{{\text{i}}} \left( {{\text{f}}_{{{\text{ij}}}} } \right)^{{2}} } \mathord{\left/ {\vphantom {{{\text{E}}_{{2}} = {\text{q}}_{{\text{i}}} \left( {{\text{f}}_{{{\text{ij}}}} } \right)^{{2}} } {\left( {{\text{E}}_{{\text{j}}} - {\text{E}}_{{\text{i}}} } \right)}}} \right. \kern-0pt} {\left( {{\text{E}}_{{\text{j}}} - {\text{E}}_{{\text{i}}} } \right)}}$$

The formula’s diagonal elements are Ej, Ei, corresponding to orbital energies and fij is off diagonal element of NBO fock Matrix and qi, which stands for the donor orbital’s occupancy. Within NBO analysis, a higher E2 signifies more pronounced interactions between donors and acceptors electron, contributing to an increased extent of conjugation within the entire system. The major donor–acceptor interactions identified through NBO analysis for the investigated compounds are represented in Tables 4, S4 and S5.

**Table 4:** Second-order perturbative analysis of donor–acceptor interactions from the Fock matrix in the NBO basis for compound (3) in its non-protonated gas-phase form.

Donnor	Type	Acceptor	Type	E_2_	Donor	Type	Acceptor	Type	E_2_
C_1_–N_2_	σ	C_1_–N_6_	σ*	0.9	C_36_–C_39_	P	N_18_–C_25_	σ*	2.33
C_1_–N_2_	σ	N_2_–C_3_	σ*	1.27	C_36_–C_39_	P	C_19_–N_24_	σ*	3.04
C_1_–N_2_	σ	C_3_–N_18_	σ*	6.41	C_1_–N_2_	P	C_3_–C_4_	p*	26.28
C_1_–N_6_	σ	C_1_–N_2_	σ*	1.0	C_1_–N_2_	P	C_5_–N_6_	p*	7.20
C_1_–N_6_	σ	C_5_–N_6_	σ*	1.01	C_3_–C_4_	P	C_1_–N_2_	p*	11.53
C_1_–N_6_	σ	C_5_–O_8_	σ*	4.81	C_3_–C_4_	P	C_5_–N_6_	p*	36.20
N_2_–C_3_	σ	C_1_–N_2_	σ*	0.9	C_3_–C_4_	P	C_19_–N_24_	p*	19.79
N_2_–C_3_	σ	C_3_–C_4_	σ*	2.05	C_5_–N_6_	P	C_1_–N_2_	p*	33.55
N_2_–C_3_	σ	C_3_–N_18_	σ*	1.45	C_5_–N_6_	P	C_3_–C_4_	p*	7.55
C_3_–C_4_	σ	N_18_–C_25_	σ*	4.69	C_19_–N_24_	P	C_3_–C_4_	p*	10.24
C_3_–C_4_	σ	C_5_–O_8_	σ*	4.38	C_25_–C_26_	P	C_28_–C_32_	p*	20.36
C_3_–C_4_	σ	C_19_–N_20_	σ*	4.69	C_28_–C_32_	P	C_27_–C_30_	p*	20.92
C_3_–N_18_	σ	C_1_–N_2_	σ*	1.62	C_27_–C_30_	P	C_25_–C_26_	p*	20.98
C_3_–N_18_	σ	N_2_–C_3_	σ*	1.74	C_27_–C_30_	P	C_28_–C_32_	p*	19.28
C_3_–N_18_	σ	C_4_–C_5_	σ*	2.06	C_28_–C_32_	P	C_25_–C_26_	p*	20.02
C_3_–N_18_	σ	N_18_–C_25_	σ*	2.14	C_28_–C_32_	p	C_27_–C_30_	p*	20.92
C_3_–N_18_	σ	C_25_–C_27_	σ*	1.27	LP1 N_2_	n	C_1_–N_6_	σ*	11.27
C_4_–C_5_	σ	C_3_–C_4_	σ*	3.06	LP1 N_2_	n	C_3_–C_4_	σ*	9.08
C_4_–C_5_	σ	C_4_–C_19_	σ*	5.47	LP1 N_2_	n	C_3_–N_18_	σ*	3.14
C_4_–C_5_	σ	O_8_–C_9_	σ*	3.54	LP1 N_6_	n	C_1_–N_2_	σ*	10.67
C_4_–C_19_	σ	N_2_–C_3_	σ*	4.95	LP1 N_6_	n	C_4_–C_5_	σ*	9.42
C_4_–C_19_	σ	C_4_–C_5_	σ*	5.31	LP1 N_6_	n	C_5_–O_8_	σ*	5.74
C_5_–N_6_	σ	C_4_–C_5_	σ*	3.14	LP1 O_8_	n	C_4_–C_5_	σ*	0.97
C_5_–N_6_	σ	C_4_–C_19_	σ*	2.61	LP1 O_8_	n	C_5_–N_6_	p*	7.33
C_9_–C_12_	σ	C_15_–O_17_	σ*	2.13	LP2 O_8_	n	C_5_–N_6_	p*	42.25
C_12_–C_15_	σ	C_15_–O_16_	σ*	1.22	LP1 O_8_	n	C_9_–C_12_	σ*	5.13
C_12_–C_15_	σ	O_17_–C_36_	σ*	3.87	LP1 O_16_	n	C_12_–C_15_	σ*	2.21
O_17_–C_36_	σ	C_12_–C_15_	σ*	2.37	LP1 O_16_	n	C_15_–O_17_	σ*	1.34
N_18_–N_24_	σ	N_2_–C_3_	σ*	3.88	LP2 O_16_	n	C_12_–C_15_	σ*	18.30
N_18_–N_24_	σ	C_19_–C_20_	σ*	3.93	LP2 O_16_	n	C_15_–O_17_	σ*	31.97
N_18_–C_25_	σ	C_3_–N_18_	σ*	2.03	LP1 O_17_	n	C_15_–O_17_	σ*	7.95
C_19_–N_24_	σ	C_4_–C_5_	σ*	3.40	LP2 O_17_	n	C_15_–O_17_	p*	44.18
C_25_–C_26_	σ	N_18_–N_24_	σ*	3.20	LP2 O_17_	n	C_36_–C_39_	p*	4.68
C_25_–C_26_	σ	C_25_–C_27_	σ*	4.53	LP1 N_18_	n	C_3_–C_4_	p*	45.95
C_26_–C_28_	σ	C_28_–C_32_	σ*	2.78	LP1 N_18_	n	C_19_–N_24_	p*	17.04
C_27_–C_30_	σ	N_18_–C_25_	σ*	3.47	LP1 N_18_	n	C_25_–C_26_	p*	28.92
C_27_–C_30_	σ	C_25_–C_27_	σ*	3.14	LP1 N_24_	n	C_3_–N_18_	σ*	5.93
C_25_–C_27_	σ	C_3_–N_18_	σ*	3.29	LP1 N_24_	n	C_4_–C_19_	σ*	5.63
C_25_–C_27_	σ	C_25_–C_26_	σ *	4.48	–	–	–	–	–
C_26_–C_28_	σ	N_18_–C_25_	σ *	3.74	–	–	–	–	–
C_26_–C_28_	σ	C_25_–C_26_	σ *	3.15	–	–	–	–	–
C_28_–C_32_	σ	C_26_–C_28_	σ *	2.63	–	–	–	–	–
C_30_–C_32_	σ	C_27_–C_30_	σ *	2.64	–	–	–	–	–

In Table 4, the perturbation energy of significant donor–acceptor interactions are comparatively presented for compound 3. The selected values represent the major overlapping between the orbitals. NBO results showed that the most important interactions in these compounds may belong to the lone pairs on O, N atoms with antibonding (C–C), (C–N) either σ or π.

In the titled compound 3, the major D-A interactions are formed between lone pair of electrons of the oxygen atom, LP (2) O_17_, which is distributed to π^*^(C_15_–O_16_) and π*(C_36_–C_37_) with stabilization energies 44.18 and 4.68 kcal/mol, respectively. Another significant electronic charge transfer is due to the donating from LP (2) O_8_ which interacts strongly with π*(C_5_–N_6_) with higher energy gain E_2_ equals to 42.25 kcal/mol. The conjugation extended via ICT from π(C_5_–N_6_) to the antibonding orbital π*(C_1_–N_2_) earning the compound 33.55 kcal/mol. This interaction decreases the electron density in (C_5_–N_6_) bond orbital and therefore, weakens the bond. The strongest possible interaction was started from LPN_18 _→ π^*^(C_3_–C_4_), π(C_3_–C_4_) → π*(C_5_–N_6_), π(C_5_–N_6_) → π* (C_1_–N_2_) with a high stabilization energy 45.95,36.20,33.55 kcal/mol respectively. The five membered ring is π excessive ring as the electron delocalization involves the interactions of LPN_18_ atom with the adjacent antibonding π^*^ (C_19_–N_24_) and π*(C_25_–C_26_) which is stabilized by 17.04 and 28.92 kcal/mol, respectively. In addition, the charge transferred from π (C_25_–C_26_), which is exemplified by three consecutive interactions as follow π (C_25_–C_26_) → π*(C_28_–C_32_), π (C_28_–C_32_) → π*( C_27_–C_30_),π ( C_27_–C_30_) → π*(C_25_–C_26_) or π*(C_28_–C_32_) with stabilization energies 20.36, 20.92, 20.98 or 19.28 kcal/mol, respectively and vice versa Table 4.These interactions make an import effect in intermolecular hyper conjugation and enhance the stability of the inhibitors. Also, the effect of LP N_2 ,_ LP N_24 ,_ LP N_6_ and sigma electrons in the charge transfer could be observed in Table 4. Furthermore, NBO analysis showed that increasing the number of lone pairs will raise the chance of different interactions, and cause further stabilization of compounds. NBO analysis for the rest compounds are in details in Tables S4, S5.

In summary, the lone pair orbitals associated with nitrogen, oxygen atoms and π electron in the inhibitors derived from pyrazolo pyrimidine exhibit hyper conjugation across all compounds. This phenomenon suggests a transfer of charge away from the heteroatoms, which corresponds to an enhancement in the effectiveness of corrosion prevention for these investigated compounds. Ultimately, the results of the theoretical calculations align with and validate the experimental findings derived from various analyses.

#### NBO atomic Charges.

The suppress of steel corrosion through inhibitor adsorption involves two mechanisms, with one of them being physical adsorption (physisorption), primarily influenced by atomic charges. Table 5, illustrates the NBO atomic charges acquired through the (6–311 +  + G (d,p)) in gas phase. The analysis of atomic charges for the examined inhibitors reveals that most of atoms have negative charge specially oxygen , nitrogen and carbon in phenyl ring, suggesting that these locations might be reasonable for the inhibitors’ interaction with the surface.

**Table 5:** The NBO natural charge for non protonated compounds in gas phase.

Atoms	Compound 1	Compound 2	Compound 3
C1	0.304	0.305	0.303
N2	− 0.577	− 0.522	− 0.578
C3	0.613	0.415	0.613
C4	− 0.232	− 0.232	− 0.232
C5	0.415	0.614	0.414
N6	− 0.520	− 0.576	− 0.522
O7	–	–	-0.532
C8	–	–	− 0.028
O8	− 0.533	− 0.533	–
C9	− 0.031	− 0.030	–
C10	–	–	–
C11	–	–	− 0.494
C12	− 0.512	− 0.501	–
C14	–	–	0.670
C15	0.806	0.815	− 0.629
N16	–	–	− 0.802
O16	− 0.599	− 0.610	–
O17	− 0.685	− 0.562	–
N18	–	− 0.228	–
C19	0.215	0.216	–
N19	–	–	− 0.228
C20	− 0.600	− 0.600	0.216
C21	–	–	− 0.601
C23	–	–	–
C24	–	–	–
N24	− 0.278	− 0.280	–
C25	–	0.159	–
N25	− 0.228	–	− 0.279
C26	0.158	− 0.226	0.158
C27	− 0.223	− 0.214	− 0.225
C28	− 0.212	− 0.188	− 0.213
C29	− 0.189	–	− 0.189
C30	–	− 0.190	–
C31	− 0.190	–	− 0.225
C32	–	− 0.215	–
C33	− 0.213	–	− 0.213
C36	–	− 0.036	–
C39	–	− 0.601	–

### FT-IR spectra of the neutral non-protonated compounds

**Fig. 7:**
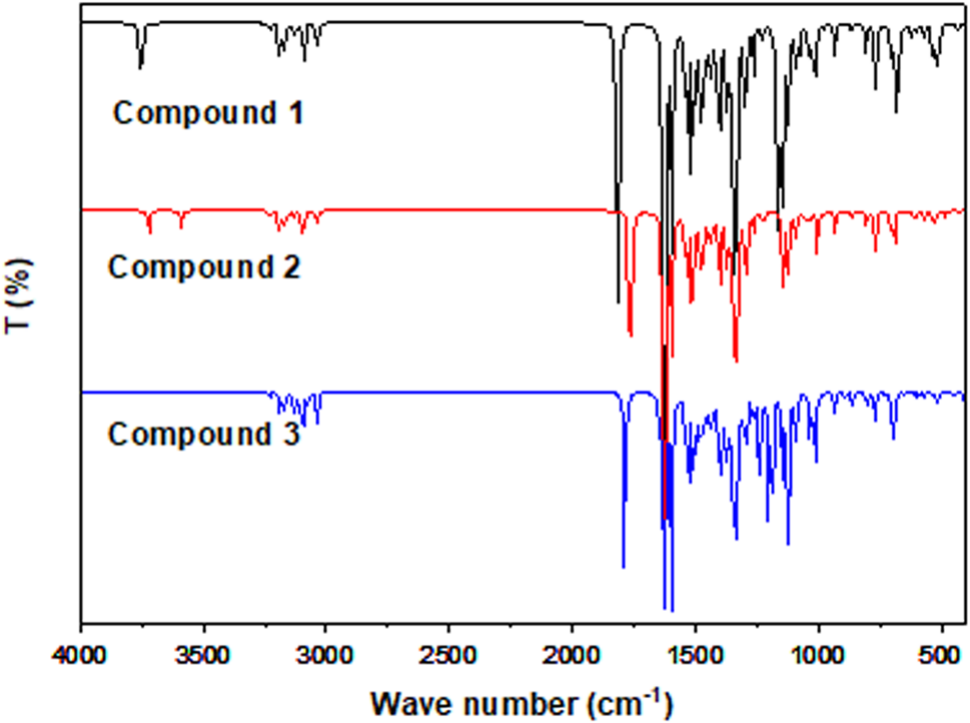
FTIR results of the investigated compounds using DFT B3LYP/6–311 +  + G(d,p). Generated by https://getintopc.com/softwares/3d-cad/originpro-2021-free-download/

IR spectrum is one of the basic properties and effective tool to identify and analyze the compounds. The FT-IR spectra of the studied compounds in gas phase were computed using harmonic force field calculation at B3LYPfunction using 6–311 +  + G(p,d) basis set. True minima on potential energy surface (PES) are represented by the optimized molecule geometries, as seen by lack of imaginary frequencies. Based on determined vibrational frequencies, their intensities, and the visualization of the vibrational modes, the FT-IR bands were assigned. IR spectra obtained from B3LYP calculations were scaled using correction factors of 0.960 and 0.961 for wave numbers above 3000 cm^−1^ and for those below 3000 cm^−1^, respectively which are standard scaling factors for the same DFT method^35^. The scaled factor is used to obtain the best agreement results with the previous studies. The calculated frequency, vibrational mode assignments and corresponding intensities for the three studied molecules are listed in Tables S6, S7 and S8. Although a wide range of vibrational bands were identified, the discussion will focus on the most significant ones, including COOH, COOEt and NH_2_. As can be seen in Fig. 7, acompound’s characteristics have been evaluated by measuring its vibrational bond.

#### NH_2_ vibrations

N–H stretching vibrations are present in all fundamental aromatic amines and typically observed in the 3000–3500 cm^−1^ region^36^. As compound 2 contains a single NH_2_ group, it exhibits two stretching vibrations. Previous studies showed that the asymmetric N–H stretching vibration appeared in the range 3422 cm^−1^^36^. Theoretical calculations gave this stretching mode with strong intensity bands at higher region 3575 cm^−1^ while the symmetric one was observed at 3245 cm^−1^ experimentally which calculated in the range 3445 cm^−1^, as shown in Table S7. The theoretical calculations showed extra vibration modes of NH_2_ as the scissoring N–H vibrations Table S7.

#### C–H vibrations

Benzene and its derivatives have C–H stretching vibrations related to the aromatic ring in the region of 3048–3197 cm^−1^^37^. The calculated frequencies of the C–H appeared at region 3038–3102 cm^−1^ for all compounds, which showed a very good agreement with the previous studies in IR spectrum^37^. Also, this spectral region contains strong –CH_2_ ass, sym. and C−H stretching vibration mode. The calculated wave numbers indicated the presence of anti-symmetric and symmetric vibrations of C−H for CH_2_ Tables S6, S7 and S8 which agrees well with the observed frequency^37^.

#### Vibrations in aromatic rings

These vibrations primarily involve complex combinations of C=C, C=N, and C–C bonds. The C=C and C–C stretches within the ring, often called semicircle stretching, produce stretching bands between 1405 and 1640 cm^−1^^38^. The C=C stretching vibrations, in particular, are observed with strong intensity at 1402 and 1660 cm^−1^. The calculations showed that the band appeared at a range of 1433–1579 cm^−1^ in the three compounds which corresponds to υC=N mode and υC–N stretching modes appeared between 1249 and 1410 cm⁻^1^ with a moderate to a strong intensity, Table S6, S7 and S8. This matches closely with the experimental ones.

#### O–H vibrations

Theoretically intensity of O–H group is found to be weaker than of N–H and characterized by very broad band which agree well with the experimental results^3^. The calculations showed very strong and broad infrared bands at 3602 were assigned to the O–H vibrations in compound 1 Table S6. Moreover, the calculations predicted the other modes of O–H group. As the O–H in plane bending of the compound were identified at 1402 cm^−1^ for compound 1 while the bands observed at 650 cm^−1^ indicate the O–H out plane bending vibrations.

#### C=O and C–O vibration

All compounds under investigation exhibit a prominent stretching band resulting from the C=O bond vibration, typically occurring within the range of 1850 to 1550 cm^3^^3^. In compound 1 the carboxylic carbonyl appeared at 1737 cm^−1^ experimentally^3^. The mode corresponding to the C=O appear at 1744 cm^−1^ with high intensity 284. In compound 2 and 3 this mode is recorded at 1716, 1741 cm^−1^, respectively. As it can be seen, there is an ethoxy group (OCH_2_CH_2_) in all compounds that having C–O bond which resulted at 1050–1361 cm^−1^. These results align well with the experimental findings.

#### Methyl group vibrations

**Fig. 8:**
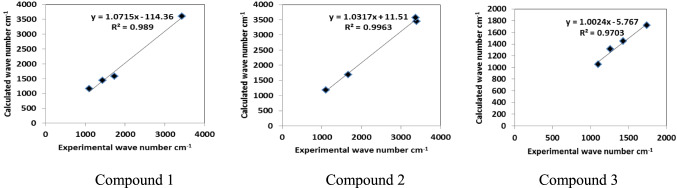
Correlation experimental and calculated wave numbers. Generated by Microsoft Excel.

In aromatic methyl for the investigated compounds, stretching vibrations of CH_3_ group (symmetric and asymmetric) are expected at 2928, 2930, 2933, 2936 cm^−1^^36^. The calculated wave numbers modes corresponding to υ_as_ CH_3_ observed at, 2914–3003 cm^−1^ compound 3. The other symmetric mode of methyl group was calculated at 2932, 2934 cm^−1^. Also, bending vibrations with CH_3_ group were appeared at a range 1343–1435 cm^−1^ in compound 3. While those for the remaining compounds are provided in Tables S6 and S7. The DFT calculations showed a good agreement with the previous studies^36,37^. Also, the correlation plots were constructed between the stimulated calculated IR spectra of the system and , experimental previous studies wave numbers^35–37^ Fig. 8. The correlation coefficient (R^2^) was calculated for each plot, where R^2^ is a statistical value of the accuracy with which the regression line approximates the actual data points. A good correlation was found as shown in Fig. 8.

### UV–visible spectra

Time-dependent DFT (TD-DFT) calculations were carried out to investigate the UV–Vis spectral properties, including the lowest energy electronic transitions and their oscillator strengths, at the B3LYP/6–311 +  + G(d,p) level of theory. Table 6 shows transition wavelength λ the oscillator strengths, vertical excitation energies, ${\varvec{f}}$ transition coefficients, and the contributions of the transitions for the main singlet–singlet transition for the three studied compounds. From the present theoretical results, six excited states, respectively, and several transitions with high percent contribution are shown in Table 6 Figs. S1, S2, S3. It is clear that the lowest electronic transition from HOMO → LUMO in the first excited state at 308.8 nm with an oscillator strength f = 0.1148, in compound 1, has the maximum contribution to the excited state 96.29%. The lowest energy electronic transition is mainly as a result of π → π* and n → π* interactions between the HOMO and LUMO orbitals. Table 6 also highlights three additional possible transitions associated with the second excited state: HOMO-1 → LUMO, HOMO → LUMO + 1, and HOMO-1 → LUMO + 3. The rest of electronic transitions for all investigated inhibitors and predominant one which are displayed in Figs. S1, S2, and S3, and Table 6.

**Table 6:** The TD-DFT calculations with main contribution to electronic excitation for non protonated investigated compounds in gas phase obtained from DFT/6-311G +  + (p,d).

Excited state	Transition	Compound 1				Compound 2				Compound 3			
		λ(nm)	${\varvec{E}}({\varvec{e}}{\varvec{V}})$	**(**${\varvec{f}})$	Cont%	λ(nm)	${\varvec{E}}({\varvec{e}}{\varvec{V}})$	(${\varvec{f}}$)	Cont%	λ(nm)	${\varvec{E}}({\varvec{e}}{\varvec{V}})$	(${\varvec{f}})$	Cont%
1	H → L	308.8	4.015	0.123	96.29	308.86	4.014	0.117	96.24	308.93	4.013	0.123	96.22
2	H − 1 → L	263.36	4.708	0.175	14.340	263.35	4.708	0.1798	13.44	263.32	4.709	0.1730	14.78
	H → L + 1				73.400				74.78				72.80
	H → L + 3				–								
3	H − 4 → L	258.81	4.791	0.705	–	258.71	4.8533	0.0606	12.05	258.45	4.797	0.0757	–
	H − 3 → L				12.47				–				6.636
	H − 1 → L				38.3				34.13				33.86
	H − 1 → L + 1				4.22				4.41				1.878
	H → L + 1				16.78				14.01				16.54
	H → L + 3				18.13				19.93				21.62
4	H − 4 → L	254.97	4.863	0.005	–	255.46	4.8533	0.02	77.82	256.03	4.843	0.014	–
	H − 3 → L				79.47				2.55				87.33
	H → L + 3				9.63				3.52				3.17
	H − 1 → L				4.37				2.10				2.42
	H − 1 → L + 1				2.77				2.01				–
	H → L + 1				–				7.46				–
5	H − 3 → L	244.73	5.066	0.0013	3.30	244.45	5.16	0.218	–	244.57	5.069	0.0025	–
	H − 2 → L				2.18				–				2.14
	H − 1 → L				38.31				40.75				40.54
	H − 1 → L + 1				14.57				13.08				13.49
	H → L + 2				36.55				3.11				4.18
	H → L + 3				–				33.77				33.38
	H − 2 → L + 1				–				2.097				2.168
6	H − 2 → L	239.36	5.17	0.2287	72.17	240.45	5.16	0.218	70.34	240.25	5.161	0.2235	70.80
	H − 2 → L + 1				4.45				4.14				4.238
	H − 1 → L				5.06				6.08				6.34
	H L + 1				3.23				3.18				3.13
	H → L + 6				8.57				6.19				6.79

### Projected density of states (PDOS) analysis

**Fig. 9:**
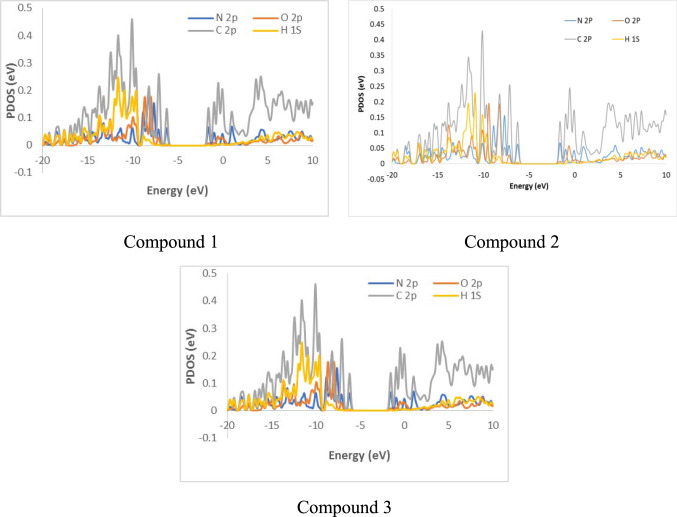
PDOS plots of the most favorable configuration of investigated compounds. Generated using GaussSum version 2.2. The software is available for download at: https://sourceforge.net/projects/gausssum/ and Microsoft excel.

PDOS analysis was carried out to examine the underlying interactions responsible for the adsorption process. The PDOS plots in Fig. 9, unveil the most energetically favorable configurations of inhibitors*⋯*Fe(110) surface. Figure 9, showed a clear overlap between the Fe(110) surface and the Cp, Op, and Np orbitals of the molecules within the valence band region, confirming the adsorption. This interaction is mainly attributed to the contribution of the C, N, and O atoms p-orbitals.

### Molecular dynamics simulations

**Fig. 10:**
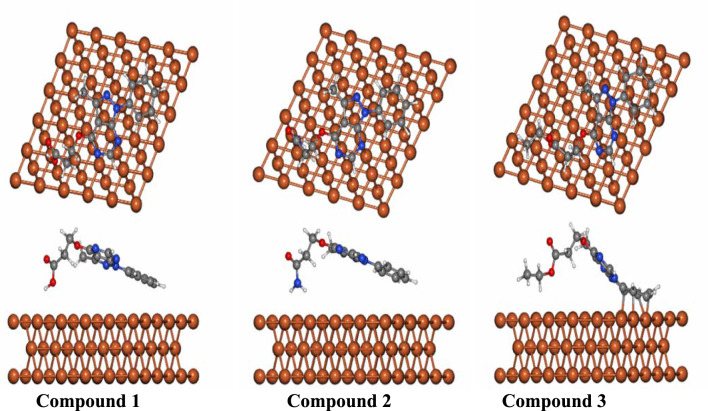
Top and side views representation of the most favorable configuration of the investigated compounds 1⋯, 2⋯, 3⋯Fe(110) surface. Generated using Quantum ESPRESSO (version 6.8; released July 19, 2021), The software can be downloaded from the official Quantum ESPRESSO website: https://www.quantum-espresso.org/

(FPMD) simulations has been proposed as an effective technique for evaluating how inhibitors adsorb onto metal surfaces^39,40^. In this study, the three target inhibitory compounds were initially positioned for adsorption onto a steel Fe (110) surface. FPMD simulations were then carried out for the investigated inhibitors and Fe (110)) complexes to evaluate the variation of total energy over simulation time. Based on the FPMD results, the most stable configuration for each complex was identified and, these optimized are presented in Fig. 10. As shown in the figure, four covalent bonds were observed in the compound 3 *⋯*Fe(110) complexes, which reflect the higher inhibition efficiency. In contrast, compounds 1 and 2 do not form any covalent bonds that may be probably due to physical adsorption. The calculated interaction energies listed in Table 7 for the inhibitor*⋯*Fe(110) complexes were all negative, indicating that adsorption occurred spontaneously. It was also evident that the compound 3*⋯*Fe(110) complex possess the highest interaction energy, reaching a value of − 129.78 eV. In alignment with quantum chemical findings, the calculated interaction energies confirmed that the inhibition efficiency followed the order 2*⋯* < 1*⋯* < 3*⋯*Fe(110).

**Table 7:** Interaction energies (E_int_(eV)) of the best configuration of the investigated inhibitors.

Systems	E_int_ (kcal/mol)	*Q*_t_ (*e*)
1	− 35.04	− 0.0884
2	− 27.91	− 0.0655
3	− 129.78	− 0.9513

This may be probably due to differences in functional groups, COOH, CONH_2_, and an COOC_2_H_5_ containing the bulky ethoxy (OC_2_H_5_) group significantly influence their adsorption behavior. The bulky ethoxy group in compound 3 is larger and has a broader surface area compared to the smaller substituents in compounds 1 and 2. This increased molecular volume of compound 3, 2797.700 bohr^3^/mol than those of compound 2, 2736.541 bohr^3^/mol) and compound 1 ,2352.589 bohr^3^/mol allows compound 3 to cover a larger area on the Fe(110) surface, enhancing van der Waals interactions and improving adsorption stability. The preference of Fe for adsorption via oxygen atoms over nitrogen atoms is mainly driven by oxygen is more electronegative, providing higher localized electron density that enhances electron donation to the metal’s d-orbitals. Oxygen’s lone pairs have better spatial and energetic alignment for strong adsorption capabilites. This is supported by the adsorption configurations shown in the Fig. 10, where oxygen-containing compounds exhibit closer and stronger affinity toward the metal surface. Also the larger and more flexible OC_2_H_5_ substituent in compound 3 facilitates a more favorable orientation during adsorption. The molecular conformation allows the oxygen atom in the ester group, which has a high electron density, to effectively come closer to the iron atoms on the surface. Additionally, the overall molecular size, distribution of active adsorption sites, and electron density around heteroatoms contribute synergistically. Substituent in compound 3 helps maintain planarity or a conformation conducive to maximizing contact with the metal surface, increasing the number of interaction points. This explains why compound 3 forms multiple covalent bonds with Fe(110), resulting in the highest interaction energy (− 129.78 eV) and superior inhibition efficiency.

### Calculations of the charge transfer

**Fig. 11:**
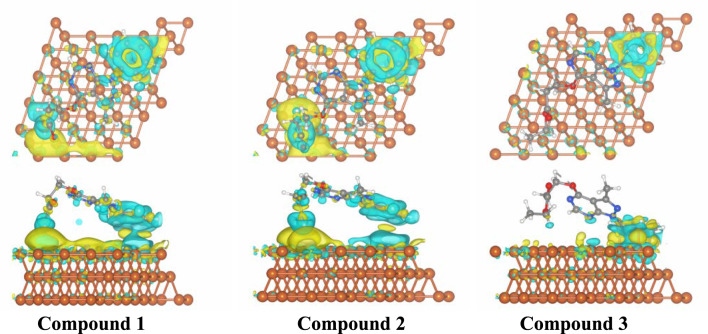
Charge Density Difference (Δρ) maps of bestconfigurations in both top and side perspectives of compound 1⋯, 2⋯, 3⋯Fe(110) complexes.Generated using VESTA software (version 3.4.4), available for download from the official site: https://www.jp-minerals.org/vesta/en/download.html

To understand charge transfer that occurs during the adsorption process, the Bader charge^41^ technique was utilized. Charge transfer values (Qt) for most stable configurations of three inhibitors *⋯*Fe(110) complexes were obtained through this method and are tabulated in Table 7. Moreover, chargedensity difference (Δρ) maps corresponding to optimal configuration showed in Fig. 11. The figure illustrates that cyan regions correspond to electron density gain, while yellow regions indicate electron density loss after adsorption, respectively. A significant accumulation of charge was observed below the three molecules, demonstrated a higher affinity for interaction with the Fe (110) surface through their oxygen atoms, nitrogen atoms, and π bond of phenyl ring. Among the three investigated compounds, compound 3 exhibits a significantly higher electron transfer to the Fe(110) surface (− 0.9513 e^−^) compared to compound 1 (− 0.0884 e^−^) and compound 2 (− 0.0655 e^−^). This difference is mainly due to the nature of the substituents present. Compound 3 contains the bulky ethoxy (OC_2_H_5_) group, which has a high localized electron density that promotes greater electron accumulation and donation to the metal surface, enhancing charge transfer during adsorption. In contrast, compounds 1 and 2 have smaller carboxylic (COOH) and amide (CONH_2_) groups, which provide less electron density and thus show lower charge transfer values. The charge density difference maps (Δρ) confirm this trend, showing more pronounced electron density gain (cyan regions) beneath compound 3, indicating stronger interaction with Fe(110). Therefore, the different substituents significantly influence the amount of charge transferred, which affects adsorption strength and corrosion inhibition efficiency. The Δρ maps for the three Fe(110) complexes were in agreement with the negative Qt values (Table 7) derived from the FPMD simulations for 1*⋯*, 2*⋯*, 3*⋯*Fe(110) complexes .

### Monte Carlo simulation

MCS is a molecular mechanics-based technique that utilizes simulated annealing for system optimization^42^. Inhibitor adsorption is primarily governed by chemical interactions, which are more significant than physical adsorption, occurring between the vacant orbitals of metal and the inhibitors electrons. The optimal adsorption for our inhibitors on carbon steel surface Fe (110) is influenced by the molecular structure and the location of charged centers within the studied compounds. The simulation results mainly provide energy parameters, as shown in Table 8.

**Table 8:** Computed Adsorption Parameters Monte Carlo Simulation.

Molecules	Total energy (kcal/mol)	Adsorptionenergy (kcal/mol)	Deformationenergy (kcal/mol)	Rigid adsorption energy (kcal/mol)	(dE_ads_/dN_i)_
1	− 171.490	− 133.142	− 19.012	− 114.130	− 133.142
2	− 180.870	− 139.003	− 23.22	− 115.883	− 139.142
3	− 192.581	− 158.998	− 10.843	− 148.15	− 158.998

**Fig. 12:**
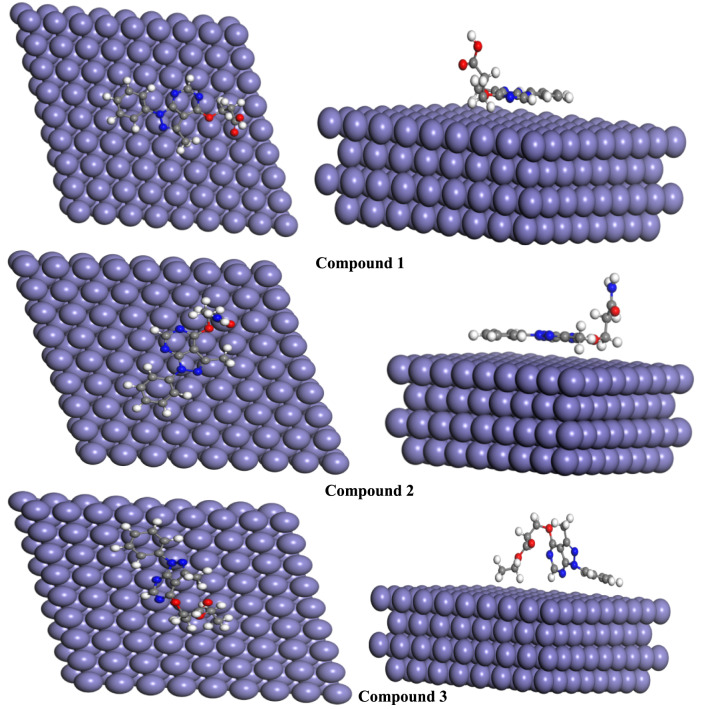
Views of the most stable low energy arrangements for the titled compounds adsorption on the surface of Fe (110) from the top and side. Generated by https://getintopc.com/softwares/simulators/biovia-materials-studio-2017-free-download/

Figure 12, represent the adsorption of the three tested compounds in the surface. Monto Carlo calculations showed that, the most reactive compound 3 with the lowest energy gap (∆E) value still has the largest adsorption energy (− 158.998 kcal mol^−1^) among the studied compounds Table 8 which could be attributed to strong connection with metal surface adsorbent. The trend of adsorption energy follows the following order 3 > 2 > 1, thus result in a great surface coverage, and hence, a greater protection indication of strong interaction between adsorbate and adsorbent.

## Conclusion

Computational chemistry plays an essential role in modern science by allowing researchers to model, predict, and understand chemical processes and adsorption on metal surface at the molecular level without the need for costly or time consuming experiments. In this work, Density functional theory was employed in order to investigate the corrosion inhibition of three pyrazole pyrimidine derivatives compound on carbon steel surface. For that purpose, adsorption modes and quantum chemical descriptors were calculated of neutral and protonated in gas and aqueous phase at B3LYP 6–311 +  + G(d,p) level of theory. The structures of protonated inhibitors give a better result than non-protonated form. The quantum chemical calculations predict compound 3 is the most effective inhibitor, because of the influence of different group substituents. The higher efficiency of compound 3 with the ethyl ester (COOEt) group followed by compound 2 with the CONH_2_ group, and then compound 1 (COOH ) is attributed to increases electron density and polarizability on the carbonyl oxygen and across the conjugated pyrazole pyrimidine system, enhancing charge transfer and adsorption strength on the Fe(110) surface. NBO interpret intermolecular and intramolecular interactions and assess the compound’s stability based on the resulting stabilization energies. Reactive sites of the investigated compounds were identified more nucleophilic and electrophilic sites on the ester derivative through Fukui function, consistent with preferential Fe-site attack. The electron localization and delocalization zones were analyzed using LOL and ELF methods. Weak van der Waals attractions and steric repulsions were identified through NCI analysis. Additionally, the simulated FTIR and TD-UV spectra of the target molecules were thoroughly examined. MCS were applied in order to find the adsorption energy. The high negative energy for adsorption energy and interaction energy demonstrates the strength of adsorption between inhibitor and surface. The obtained results from FPMD simulation agree well with Monte Carlo and quantum chemical parameters. A distinct correlation was observed between interaction energies and quantum descriptors. This mainly due to influence of substituent as compound 3 with ethyl ester (COOEt) group, which demonstrated large volume, hydrophobic and bulk character also improve adsorption orientation and surface coverage. PDOS plots confirmed the adsorption of the studied inhibitors on the **Fe(110)** surface . Generally, the inhibition efficiency of the studied compounds increased in the order: 1 < 2 < 3. Finally, this is a good agreement between the theoretical investigations, which showed the reliability of the level of calculation employed in this study.

## Computational method

Density Functional Theory (DFT) calculations was performed on pyrazolo pyrimidine derivatives in both gas and aqueous phases, with the B3LYP method and 6–311 +  + G(d,p) basis set^43,44^ with the help of Gaussian 03 software package^45^. This method has gained popularity in recent years due to its ability to provide accurate results in a relatively short amount of time^43^. The inhibition efficiency of the investigated compounds^3^ was evaluated in the aqueous medium using the polarizable continuum model, which treated the investigated molecules as trapped in a cavity of the water solvent. The IEFPCM version of PCM was utilized^46^. Geometrical optimization was initially employed for the inhibitors, then by frequency calculations to confirm that the obtained structures represented true minima as no imaginary frequencies were detected. Visualization of the molecular orbital HOMO, and LUMO distribution of the studied compounds was carried out using Gauss View^47^. According to Koopman equation^16^

$$\begin{aligned} E_{gap} = & E_{LUMO} - E_{HOMO} \\ IP = & - E_{HOMO} \\ \eta = & {{\left( {IP{-}EA} \right)} \mathord{\left/ {\vphantom {{\left( {IP{-}EA} \right)} 2}} \right. \kern-0pt} 2} \\ \sigma = & {1 \mathord{\left/ {\vphantom {1 \eta }} \right. \kern-0pt} \eta } \\ \chi = & IP + EA/{2} \\ \mu = & - \chi \\ \omega = & \mu^{{2}} /{2}\eta \\ \end{aligned}$$

The number of electron transfer ΔN_Max_ = [Φ_Metal_ − *X*_Inhibitor_] / 2(*η*_Metal_ + *η*_Inhibitor_) where the parameters Φ_Metal_, *X*_Inhibitor_, η_Metal_, and η_Inhibitor_ reflect the metal’s work function, the inhibitor’s electronegativity and the hardness of both the metal and the inhibitor, respectively. As suggested in the previous studies, the values for Φ_Metal_ and η_Metal_ were taken to be 5.07 eV and 0 eV, respectively^18^. The energy of back donation is calculated as Δ*E*_Backdonation_ =  − *η*/4^20^.To examine local reactivity of molecules, the Fuki function indix, and their difference (Δf(r)) dual descriptor were evaluated. NBO analysis, TD- DFT and FT-IR has been done using the same method. LOL, ELF, were plotted using calculations carried out with Multiwave function^23^ based on a formatted checkpoint (fch) file obtained from Gaussian^45^ , while NCI visualization occurring using Multiwfn^23^ and Visual Molecular Dynamics (VMD)^27^.

### Monte Carlo simulation

Monte Carlo (MC) simulations were conducted using BIOVIA Materials Studio 2017^48^. Prior to running the MC simulations, the clean Fe (110) surface while the studied molecules, and the surrounding environment were optimized using the Forcite module. Adsorption Locator module^49^ was then applied to identify possible adsorption configurations via Monte Carlo simulated annealing. All simulations were performed using COMPASS (Condensed-phase Optimized Molecular Potentials for Atomistic Simulation Studies) force field^50^.

### Molecular dynamic simulation

Using Quantum ESPRESSO version 6.4.1, (FPMD) were used to examine the inhibitors’ adsorption properties on the metallic surface^51,52^.The simulations utilized the Generalized Gradient Approximation (GGA) for the exchange–correlation functional, specifically adopting the Perdew-Burke-Ernzerhof (PBE) formulation^53^. Electronion interactions were described using ultra soft pseudo potentials^54^. Plane-wave basis sets were conducted to represent Kohn–Sham orbitals and charge densities, with kinetic energy cutoffs set to 50 Ry and 500 Ry, respectively. To account for long-range dispersion interactions, Grimme’s DFT-D2 method was applied throughout the calculations^55^. Convergence thresholds were defined as 10^−4^ eV for energy and 10^−3^ eV/Å for forces. The Marzari–Vanderbilt smearing technique was implemented to handle partial electron occupations^56^. The simulations involved over 1000 steps with time step of 0.97 femtoseconds using the Verlet algorithm to monitor the movement of electrons and atoms at each interval^57^.

### Calculations of interactions and binding energies.

 Following their individual FPMD simulations, the three inhibitor complexes’ most stable structures were chosen and then put through relaxation calculations. The Brillouin zone was sampled using a 2 × 2 × 1 k-point grid. During the relaxation, the Fe atoms in the bottom layer remained fixed. In contrast to those in the upper two layers, which were completely tuned to achieve stability, the interaction (E_int_) energies were computed using the following formulas:

E_int_ = E_inhibitor_*⋯*_Fe(110)_ − (E_Fe(110)incomplex_ + E_inhibitorincomplex_) where E_inhibitor_*⋯*_Fe(110),_ E_Fe(110)incomplex_ E_inhibitorincomplex_ E_Fe(110),_ E_inhibitor_ represent the energies of the complex, the Fe(110) surface within the complex, the inhibitors within the complex, , respectively. Additionally, the Bader charge method^41^ was applied to determine the charge transfer (Q_t_) to or from the inhibitors using the formula:

Q_t_ = Q_adsorbedinhibitor − _Q_isolatedinhibitor_ where the *Q* adsorbed inhibitor and *Q* isolated inhibitor are the charges of the inhibitor when adsorbed onto the surface and when isolated, respectively The following equation was used to represent the charge density difference (Δρ).

Δ*ρ* = *ρ*_inhibitor_*⋯*_Fe (110)_ − *ρ*_Fe(110) − _*ρ*_inhibitor_ where the *ρ*_inhibitor_*⋯*_Fe (110)_, *ρ*_Fe (110)_, and *ρ*_inhibitor_ are the, the complex’s charge densities, the Fe(110) surface charge densities and adsorbed inhibitors’ charge densities. VESTA visualization software was used to show the charge density graphs^58^. Additionally, a projected density of states (PDOS) analysis was also carried out.

## Supplementary Information

Below is the link to the electronic supplementary material.

Supplementary Material 1

## Data Availability

The data sets generated and/or analyzed during the current study are provided as supplementary files “Data Availability”.
