# Supplementary material for: A multiscale computational investigation for protection of carbon steel surface by pyrazolo-pyrimidine derivatives
Source: Sci Rep. 2025 Sep 17;15:32576. doi: 10.1038/s41598-025-19022-6 (PMC12443998; doi:10.1038/s41598-025-19022-6)
Supplement: Supplementary file 1 — Supplementary Material 1 [file 41598_2025_19022_MOESM1_ESM.docx]

**Authors and affiliations**

**Mohamed K. Awad**

Chemistry Department, Theoretical Applied Chemistry Unit (TACU)

Faculty of Science, Tanta University

Tanta, Egypt

**W.S. Abdel Halim**

Chemistry Department,

Faculty of Science, Zagazig University

Zagazig, Egypt

**Faten M. Atlam**

Chemistry Department, Theoretical Applied Chemistry Unit (TACU)

Faculty of Science, Tanta University

Tanta, Egypt

**Mohamed M. Fawzy**

Chemistry Department

Faculty of Science, Zagazig University, Egypt

Zagazig, Egypt

**Correspondence:** mohamed.awad1@science.tanta.edu.eg

**Supplementary Tables**

**Table** **S1.** The condensed Fuki function and local softness for compound **1**.

| ∆S | (*f -* **) σ** | (*f +*) **σ** | ∆ *f* | *f* - | *f +* | Atom number |
| --- | --- | --- | --- | --- | --- | --- |
| 0.047 | -0.008 | -0.063 | -0.128 | -0.019 | -0.147 | C1 |
| -0.042 | 0.029 | -0.013 | -0.037 | 0.068 | -0.031 | N2 |
| 0.015 | 0.000 | 0.015 | 0.035 | 0.000 | 0.035 | C3 |
| -0.021 | -0.017 | -0.013 | 0.009 | -0.040 | -0.031 | C4 |
| -0.010 | 0.006 | 0.023 | 0.038 | 0.015 | 0.053 | C5 |
| -0.016 | -0.019 | -0.022 | -0.007 | -0.044 | -0.052 | N6 |
| -0.005 | -0.008 | -0.011 | -0.007 | -0.018 | -0.025 | O8 |
| 0.027 | 0.006 | -0.014 | -0.048 | 0.015 | -0.033 | C9 |
| -0.174 | 0.003 | 0.180 | 0.412 | 0.008 | 0.419 | C12 |
| -0.062 | -0.001 | 0.060 | 0.142 | -0.002 | 0.140 | C15 |
| -0.002 | -0.006 | -0.009 | -0.009 | -0.013 | -0.022 | O16 |
| 0.007 | -0.003 | -0.012 | -0.022 | -0.006 | -0.028 | O17 |
| -0.008 | -0.010 | -0.012 | -0.005 | -0.023 | -0.028 | C19 |
| -0.020 | -0.006 | 0.007 | 0.032 | -0.014 | 0.017 | C20 |
| 0.017 | -0.014 | -0.046 | -0.073 | -0.033 | -0.106 | N24 |
| -0.034 | 0.036 | 0.002 | -0.079 | 0.084 | 0.005 | N25 |
| -0.016 | 0.006 | 0.028 | 0.052 | 0.015 | 0.066 | C26 |
| -0.020 | -0.014 | -0.008 | 0.014 | -0.033 | -0.018 | C27 |
| -0.023 | -0.021 | -0.019 | 0.004 | -0.049 | -0.045 | C28 |
| -0.013 | -0.013 | -0.014 | -0.001 | -0.031 | -0.032 | C29 |
| -0.018 | -0.015 | -0.011 | 0.008 | -0.034 | -0.026 | C31 |
| -0.046 | -0.029 | -0.011 | 0.041 | -0.067 | -0.026 | C33 |

**Table S2.** The condensed Fuki function and local softness for compound **2**.

| ∆S | (*f -* **) σ** | (*f +*) **σ** | ∆ *f* | *f* - | *f +* | Atom number |
| --- | --- | --- | --- | --- | --- | --- |
| -0.058 | -0.008 | -0.065 | -0.133 | -0.018 | -0.152 | C1 |
| -0.018 | -0.003 | -0.015 | -0.028 | -0.007 | -0.035 | N2 |
| 0.045 | 0.001 | 0.047 | 0.104 | 0.003 | 0.108 | C3 |
| -0.015 | -0.018 | -0.033 | -0.034 | -0.042 | -0.077 | C4 |
| -0.001 | 0.005 | 0.004 | -0.003 | 0.012 | 0.009 | C5 |
| 0.001 | -0.019 | -0.017 | 0.002 | -0.043 | -0.040 | N6 |
| -0.006 | -0.008 | -0.014 | -0.014 | -0.018 | -0.033 | O7 |
| -0.055 | 0.007 | -0.048 | -0.127 | 0.016 | -0.111 | C8 |
| 0.232 | 0.007 | 0.239 | 0.539 | 0.016 | 0.555 | C11 |
| 0.036 | -0.004 | 0.031 | 0.083 | -0.010 | 0.073 | C14 |
| 0.001 | -0.013 | -0.012 | 0.002 | -0.030 | -0.028 | O15 |
| 0.016 | 0.003 | -0.013 | -0.036 | 0.006 | -0.030 | N16 |
| 0.032 | -0.031 | 0.001 | 0.074 | -0.072 | 0.002 | N19 |
| 0.029 | -0.008 | 0.021 | 0.067 | -0.019 | 0.048 | C20 |
| 0.003 | -0.006 | -0.003 | 0.008 | -0.014 | -0.006 | C21 |
| -0.028 | -0.014 | -0.042 | -0.066 | -0.032 | -0.098 | N25 |
| 0.002 | 0.009 | 0.011 | 0.005 | 0.020 | 0.025 | C26 |
| 0.013 | -0.014 | -0.001 | 0.031 | -0.033 | -0.003 | C27 |
| -0.001 | -0.019 | -0.021 | -0.003 | -0.045 | -0.048 | C28 |
| 0.003 | -0.014 | -0.012 | 0.006 | -0.033 | -0.027 | C29 |
| 0.005 | -0.016 | -0.010 | 0.012 | -0.036 | -0.024 | C31 |
| 0.019 | -0.029 | -0.009 | 0.045 | -0.067 | -0.022 | C33 |

**Table S3.** The condensed Fuki function and local softness of compound **3**.

| ∆S | (*f -* **) σ** | (*f +*) **σ** | ∆ *f* | *f* - | *f +* | Atom number |
| --- | --- | --- | --- | --- | --- | --- |
| 0.006 | -0.005 | 0.001 | 0.034 | -0.011 | 0.023 | C1 |
| -0.077 | -0.019 | -0.096 | -0.179 | -0.044 | -0.223 | N2 |
| -0.209 | 0.005 | -0.204 | -0.485 | 0.011 | -0.474 | C3 |
| 0.443 | -0.019 | 0.424 | 1.029 | -0.044 | 0.985 | C4 |
| -0.179 | 0.000 | -0.179 | -0.416 | 0.001 | -0.415 | C5 |
| -0.120 | -0.028 | -0.148 | -0.279 | -0.066 | -0.345 | N6 |
| -0.029 | 0.006 | -0.023 | -0.041 | 0.013 | -0.054 | O8 |
| -0.164 | 0.008 | -0.156 | -0.382 | 0.019 | -0.363 | C9 |
| 0.019 | 0.007 | 0.026 | 0.044 | 0.016 | 0.060 | C12 |
| -0.142 | -0.002 | -0.144 | -0.329 | -0.005 | -0.335 | C15 |
| -0.111 | -0.004 | -0.115 | -0.256 | -0.010 | -0.267 | O16 |
| 0.004 | 0.000 | 0.005 | 0.010 | 0.001 | 0.011 | O17 |
| -0.220 | -0.032 | -0.253 | -0.512 | -0.075 | -0.587 | N18 |
| -0.519 | 0.506 | -0.013 | -1.207 | 1.177 | -0.030 | C19 |
| -0.296 | -0.006 | -0.302 | -0.689 | -0.013 | -0.702 | C20 |
| 0.080 | -0.014 | 0.067 | 0.188 | -0.032 | 0.155 | N24 |
| -0.002 | 0.012 | 0.010 | -0.004 | 0.028 | 0.024 | C25 |
| 0.038 | -0.015 | 0.023 | 0.090 | -0.035 | 0.054 | C26 |
| 0.022 | -0.018 | 0.004 | 0.051 | -0.041 | 0.010 | C27 |
| -0.188 | -0.015 | -0.203 | -0.437 | -0.035 | -0.472 | C28 |
| -0.172 | -0.018 | -0.189 | -0.399 | -0.041 | -0.440 | C30 |
| -0.200 | -0.031 | -0.231 | -0.464 | -0.072 | -0.536 | C32 |
| -0.207 | -0.006 | -0.213 | 0.480 | -0.014 | -0.494 | C36 |
| 0.102 | 0.003 | 0.105 | 0.238 | 0.006 | 0.243 | C39 |

**Table S4.** Second-order perturbative analysis of donor–acceptor interactions from the Fock matrix in the NBO basis for compound (**1**) in its non-protonated gas-phase form.

| **Donnor** | **Type** | **Acceptor** | **Type** | **E_2_** | **Donor** | **Type** | **Acceptor** | **Type** | **E_2_** |
| --- | --- | --- | --- | --- | --- | --- | --- | --- | --- |
| C_1_-N_2_ | σ | C_1_-N_6_ | σ* | 1.38 | C_26_-C_27_ | π | C_23_-C_24_ | π* | 16.57 |
| C_1_-N_2_ | σ | N_2_-C_3_ | σ* | 1.35 | C_26_-C_27_ | π | C_5_- N_25_ | π* | 8.64 |
| C_1_-N_6_ | σ | C_1_-N_2_ | σ* | 1.0 | C_26_-C_27_ | π | C_28_-C_31_ | π* | 19.03 |
| C_1_-N_6_ | σ | C_5_-N_6_ | σ* | 1.27 | C_26_-C_27_ | π | C_29_-C_33_ | π* | 20.04 |
| C_1_-N_6_ | σ | C_5_- N_25_ | σ* | 6.34 | C_28_-C_31_ | π | C_26_-C_27_ | π* | 21.02 |
| N_2_-C_3_ | σ | C_3_-C_4_ | σ* | 3.16 | C_28_-C_31_ | π | C_29_-C_33_ | π* | 19.05 |
| N_2_-C_3_ | σ | C_4_-C_19_ | σ* | 2.16 | C_29_-C_33_ | π | C_26_-C_27_ | π* | 20.07 |
| C_3_-C_4_ | σ | C_4_-C_5_ | σ* | 3.05 | C_29_-C_33_ | π | C_28_-C_31_ | π* | 19.81 |
| C_3_-C_4_ | σ | C_4_-C_19_ | σ* | 5.49 | LP1 N_2_ | n | C_1_-N_6_ | σ* | 10.64 |
| C_4_-C_5_ | σ | C_3_-O_8_ | σ* | 4.42 | LP1 N_2_ | n | C_3_-C_4_ | σ* | 9.40 |
| C_4_-C_5_ | σ | N_25_-C_26_ | σ* | 5.50 | LP1 N_2_ | n | C_3_-O_8_ | σ* | 5.78 |
| C_4_-C_19_ | σ | C_3_-C_4_ | σ* | 5.32 | LP1 N_6_ | n | C_1_-N_2_ | σ* | 11.30 |
| C_19_-N_24_ | σ | C_3_- C_4_ | σ* | 3.40 | LP1 N_6_ | n | C_4_-C_5_ | σ* | 9.13 |
| N_24_-N_25_ | σ | C_5_  -N_6_ | σ* | 3.88 | LP1 N_6_ | n | C_5_-N_25_ | σ* | 3.10 |
| N_24_-N_25_ | σ | C_5_-N_25_ | σ* | 1.05 | LP1 N_24_ | n | C_4_-C_19_ | σ* | 5.62 |
| N_24_-N_25_ | σ | C_19_-C_20_ | σ* | 4.06 | LP1 N_24_ | n | C_5_-N_25_ | σ* | 5.88 |
| N_25_-C_26_ | σ | C_4_-C_5_ | σ* | 0.73 | LP1 N_24_ | n | C_19_-C_20_ | σ* | 0.69 |
| N_25_- C_26_ | σ | C_5_- N_25_ | σ* | 2.03 | LP1 O_8_ | n | N_2_-C_3_ | σ* | 7.26 |
| N_25_-N_26_ | σ | C_19_ -N_24_ | σ* | 1.29 | LP1 O_8_ | n | C_3_-C_4_ | σ* | 0.96 |
| C_26_-C_27_ | σ | N_24_-N_25_ | σ* | 3.16 | LP2 O_8_ | n | N_2_-C_3_ | π* | 41.74 |
| C_26_-C_27_ | σ | C_26_-C_28_ | σ* | 4.55 | LP2 O_8_ | n | C_9_- C_12_ | π* | 5.25 |
| C_26_-C_27_ | σ | C_5_-N_25_ | σ* | 8.64 | LP1 O_16_ | n | C_12_- C_15_ | σ* | 2.33 |
| C_26_-C_27_ | σ | C_28_-C_31_ | σ* | 19.03 | LP1 O_16_ | n | C_15_- C_17_ | σ* | 1.36 |
| C_26_-C_27_ | σ | C_29_-C_33_ | σ* | 20.04 | LP2 O_16_ | n | C_12_- C_15_ | π* | 18.27 |
| C_26_-C_28_ | σ | C_5_-N_25_ | σ* | 3.22 | LP2 O_16_ | n | C_15_- O_17_ | π* | 33.37 |
| C_26_-C_28_ | σ | C_26_-C_27_ | σ* | 4.51 | LP1O_17_ | n | C_15_- O_16_ | σ* | 6.45 |
| C_27_-C_29_ | σ | N_25_-C_26_ | σ* | 4.37 | LP2O_17_ | n | C_15_- O_16_ | π* | 38.09 |
| C_27_-C_29_ | σ | C_26_-C_27_ | σ* | 3.15 |  |  |  |  |  |
| C_27_-C_29_ | σ | C_29_-C_33_ | σ* | 2.33 |  |  |  |  |  |
| C_28_-C_31_ | σ | N_25_-C_26_ | σ* | 4.13 |  |  |  |  |  |
| C_28_-C_31_ | σ | C_26_-C_28_ | σ* | 3.14 |  |  |  |  |  |
| C_1_-N_6_ | π | N_2_-C_3_ | π* | 7.22 |  |  |  |  |  |
| C_1_-N_6_ | π | C_5_-N_25_ | π* | 37.27 |  |  |  |  |  |
| N_2_-C_3_ | π | C_1_-N_6_ | π* | 33.20 |  |  |  |  |  |
| C_3_-C_4_ | π | C_1_- N _2_ | π* | 31.85 |  |  |  |  |  |
| C_3_-C_4_ | π | C_19-_ N_24_ | π* | 19.79 |  |  |  |  |  |
| C_3_-C_4_ | π | C_5_ - N_6_ | π* | 36.02 |  |  |  |  |  |
| C_5_- N_25_ | π | C_1_-N_6_ | π* | 5.13 |  |  |  |  |  |
| C_5_- N_25_ | π | C_26_-C_27_ | π* | 13.16 |  |  |  |  |  |
| C_5_- N_25_ | π | C_19_- N_24_ | π* | 5.13 |  |  |  |  |  |
| C_19_- N_24_ | π | C_5_- N_25_ | π* | 8.27 |  |  |  |  |  |

**Table S5.** Second-order perturbative analysis of donor–acceptor interactions from the Fock matrix in the NBO basis for compound (**2**) in its non-protonated gas-phase form.

| **Donnor** | **Type** | **Acceptor** | **Type** | **E_2_** | **Donor** | **Type** | **Acceptor** | **Type** | **E_2_** |
| --- | --- | --- | --- | --- | --- | --- | --- | --- | --- |
| C_1_-N_2_ | σ | C_1_-N_6_ | σ* | 1.00 | C_1_-N_6_ | π | N_2_-C_3_ | π* | 7.14 |
| C_1_-N_2_ | σ | N_2_-C_3_ | σ* | 0.98 | C_1_-N_6_ | π | C_5_-N_19_ | π* | 37.25 |
| C_1_-N_6_ | σ | C_3_ – O_7_ | σ* | 4.83 | N_2_-C_3_ | π | C_1_-N_6_ | π* | 33.45 |
| C_1_-N_6_ | σ | C_5_-N_19_ | σ* | 6.47 | C_5_- N_19_ | π | C_1_-N_6_ | π* | 9.51 |
| C_1_-N_6_ | σ | C_29_-C_33_ | σ* | 0.81 | C_5_- N_19_ | π | C_20_ - N _25_ | π* | 24.01 |
| N_2_-C_3_ | σ | N_2_-C_3_ | σ* | 7.14 | C_5_- N_19_ | π | C_26_ - C_27_ | π* | 22.62 |
| N_2_-C_3_ | σ | C_1_ - N _2_ | σ* | 0.92 | C_5_- N_19_ | π | C_29_ - C_33_ | π* | 17.07 |
| N_2_-C_3_ | σ | C_3_-C_4_ | σ* | 3.13 | C_20_ - N_25_ | π | C_5_- N_19_ | π* | 8.45 |
| N_2_-C_3_ | σ | C_4_- C_20_ | σ* | 2.60 | C_26_ - C_27_ | π | C _5_ - N _19_ | π* | 8.69 |
| C_3_-C_4_ | σ | N_2_-C_3_ | σ* | 2.85 | C_26_ -C_27_ | π | C _28_ – C_31_ | π* | 19.14 |
| C_3_-C_4_ | σ | C_3_ – O_7_ | σ* | 1.21 | C_26_ - C_27_ | π | C_29_ - C _33_ | π* | 18.88 |
| C_3_-C_4_ | σ | C_4_-C_5_ | σ* | 3.05 | C_28_ - C_31_ | π | C_26_ - C _27_ | π* | 21.07 |
| C_3_-C_4_ | σ | C_4_ - C _20_ | σ* | 5.46 | C_28_-C_31_ | π | C_29_-C_33_ | π* | 20.41 |
| C_3_ - O_7_ | σ | C_1_ - N_2_ | σ* | 1.92 | C_29_-C_33_ | π | C_26_ - C _27_ | π* | 20.15 |
| C_3_ - O_7_ | σ | N _2_ - C_3_ | σ* | 0.79 | C_29_-C_33_ | π | C_28_ - C_31_ | π* | 20.97 |
| C_3_ - O_7_ | σ | C_4_ - C_5_ | σ* | 1.04 | LP1 N_2_ | n | C_1_-N_6_ | σ* | 10.70 |
| C_4_ - C_5_ | σ | C_3_-C_4_ | σ* | 2.74 | LP1 N_2_ | n | C_3_-C_4_ | σ* | 9.42 |
| C_4_ - C_5_ | σ | C _3_ - O_7_ | σ* | 4.35 | LP1 N_2_ | n | C_3_-O_7_ | σ* | 5.67 |
| C_4_ - C_5_ | σ | C _4_ - C_20_ | σ* | 3.05 | LP1 N_6_ | n | C_1_-N_2_ | σ* | 11.42 |
| C_4_ - C_5_ | σ | N_19_ - C_26_ | σ* | 5.49 | LP1 N_6_ | n | C_4_-C_5_ | σ* | 9.11 |
| C_4_ - C_5_ | σ | C_20_- C_21_ | σ* | 4.39 | LP1 N_6_ | n | C_5_-N_19_ | σ* | 3.17 |
| C_4_ – C_20_ | σ | C_3_ - C_4_ | σ* | 5.31 | LP1 N_16_ | n | C_14_-O_15_ | σ* | 2.22 |
| C_4_ – C_20_ | σ | C _5_ - N_6_ | σ* | 5.04 | LP1 N_16_ | n | C_14_-O_15_ | π* | 42.75 |
| C_4_ – C_20_ | σ | C_20_ – C_21_ | σ* | 1.91 | LP1 N_25_ | n | C_4_-C_20_ | σ* | 5.62 |
| C_5_ - N_6_ | σ | C_4_ - C_5_ | σ* | 2.85 | LP1 N_25_ | n | C_5_ - N _19_ | σ* | 5.95 |
| C_5_ - N_6_ | σ | N_19_-N_25_ | σ* | 1.27 | LP1 O_7_ | n | N_2_-C_3_ | σ* | 7.42 |
| C_5_ – N_19_ | σ | C_3_- C_4_ | σ* | 2.05 | LP2 O_7_ | n | N_2_-C_3_ | π* | 42.79 |
| C _8_ - C_11_ | σ | C_14_ – O_15_ | σ* | 1.01 | LP2 O_7_ | n | C _8_ - C _11_ | π* | 5.12 |
| C _8_ - C_11_ | σ | N_14_ - N_16_ | σ* | 1.87 | LP1 O_15_ | n | C_11_- C_14_ | σ* | 2.14 |
| C_11_ - C_14_ | σ | C_14_ – O_15_ | σ* | 0.90 | LP1 O_15_ | n | C_11_- C_14_ | π* | 19.61 |
| C_14_ - C_15_ | σ | C_5_ - N_6_ | σ* | 1.71 | LP2O_15_ | n | C_14_ - N_16_ | σ* | 1.55 |
| C_14_ - C_15_ | σ | C_11_ – C_14_ | σ* | 1.93 | LP2 O_15_ | n | C_14_ - N_16_ | π* | 25.12 |
| N_19_- C_25_ | σ | C_5_- N_6_ | σ* | 3.86 |  |  |  |  |  |
| N_19_- C_25_ | σ | C_20_- C_21_ | σ* | 4.12 |  |  |  |  |  |
| N_19_- C_25_ | σ | C_20_ - N_25_ | σ* | 1.29 |  |  |  |  |  |
| C_20_ - C _21_ | σ | N_19_ – N_25_ | σ* | 2.68 |  |  |  |  |  |
| C_20_ - C _21_ | σ | C_20_ - N_25_ | σ* | 1.87 |  |  |  |  |  |
| C_20_ - N_25_ | σ | C_3_-C_4_ | σ* | 3.43 |  |  |  |  |  |
| C_20_ - N_25_ | σ | N_19_ **-** C _26_ | σ* | 3.68 |  |  |  |  |  |
| C_26_ - C_27_ | σ | N_19_ – N | σ* | 3.18 |  |  |  |  |  |
| C_26_ - C_27_ | σ | C_26_ - C_28_ | σ* | 4.55 |  |  |  |  |  |
| C_26_ - C_28_ | σ | C_5_ – N_19_ | σ* | 3.26 |  |  |  |  |  |
| C_27_ - C_29_ | σ | N_19_ - C_26_ | σ* | 4.37 |  |  |  |  |  |

**Table S6.** Observed and calculated vibrational frequency of compound **(1**) at B3LYP method with 6-311+G(d,p).

| Mode | Compound 1 | | | | |
| --- | --- | --- | --- | --- | --- |
|  | Assignment | Exp | Cal | Scale | I IR |
| 20 | γ (C- C) |  | 416 | 400 | 0.083 |
| 21 | γ (C- C-C) |  | 433 | 416 | 7.80 |
| 22 | γ (C- C-C) |  | 509 | 489 | 1.61 |
| 23 | γ (C- C-C)+ deformation γ (C- O-C) |  | 514 | 494 | 44.48 |
| 24 | Out of the plane bending CO + deformation (C- O-C) , |  | 530 | 509 | 37 |
| 25 | δ (C-C-N) |  | 535 | 514 | 5.62 |
| 26 | δ (C-C-N) |  | 570 | 548 | 10.47 |
| 27 | W (O-C-N-C) + t( HCCC) |  | 601 | 578 | 9 |
| 28 | Out of the plane bending CO |  | 622 | 598 | 17.11 |
| 29 | In the plane (C- C-C)ph |  | 632 | 607 | 0.89 |
| 30 | In the plane (C- C-C)ph + N-N |  | 650 | 625 | 0.49 |
| 31 | In the plane (C- C-C) pyraz+ |  | 656 | 630 | 0.8 |
| 32 | δ (C- C-N) |  | 676 | 650 | 108 |
| 33 | In the plane (C- C-C)ph + δ (C-C-N) |  | 689 | 662 | 36.38 |
| 34 | In the plane (C- C-C)ph + δ (C-C-N) |  | 695.8 | 669 | 7.15 |
| 35 | In the plane (C- C-C)ph **+opb(C-H)Ar** |  | 704.8 | 677 | 23.10 |
| 36 | opb(C-H)Ar+δ(C-H)CH_2_ |  | 727.97 | 700 | 1.754 |
| 37 | opb(C-H)Ar |  | 768.5 | 739 | 50 |
| 38 | opb(C-H)Ar+δ(C-H)CH_2_ |  | 803 | 772 | 19 |
| 39 | opb(C-H)Ar |  | 808 | 776 | 8.87 |
| 40 | υ(C-O)+opb(C-H)Ar+δ(C-H)CH_2_ |  | 854 | 821 | 0.144 |
| 41 | opb(C-H)Ar |  | 864 | 830 | 9.15 |
| 42 | opb(C-H)Ar+δ(C-H)CH_2_ |  | 933 | 897 | 6.5 |
| 43 | opb(C-H)Ar |  | 934 | 898 | 28.9 |
| 44 | υ (C=N) +opb(C-H)Ar+δ(C-H)CH_2_ |  | 978 | 940 | 3.78 |
| 45 | υ (C=N) +opb(C-H)pyri |  | 991 | 952 | 0.46 |
| 46 | opb(C-H)Ar |  | 999 | 960 | 0.54 |
| 47 | opb(C-H)Ar |  | 1001 | 962 | 15.325 |
| 48 | δ(C-H)CH_2_ |  | 1014 | 974 | 30.65 |
| 49 | Ipb (C-H) CH_3_+opb(C-H)Ar+δ(C-H)CH_2_ |  | 1018 | 978 | 35.17 |
| 50 | Ipb (C-H) CH_3_+opb(C-H)Ar+δ(C-H)CH_2_ |  | 1035 | 995 | 39 |
| 51 | Ipb (C-H) CH_3_ |  | 1050 | 1009 | 0.88 |
| 52 | Ipb (C-H)+ δ(C-H)CH_2_ |  | 1059 | 1018 | 0.83 |
| 53 | Ipb (C-H)+ δ(C-H)CH_2_ |  | 1078 | 1036 | 36.12 |
| 54 | υ (C-O)+ δ(C-H)CH_2_ |  | 1093 | 1050 | 19 |
| 55 | υ (N-N)+ Ipb (C-H)+ δ(C-H)CH_2_ |  | 1094 | 1051 | 8.35 |
| 56 | υ (C=N) + Ipb (C-H)Ar+ (C-H)CH_2_ |  | 1124 | 1080 | 161.1 |
| 57 | .(N-N)+ Ipb (C-H)Ar+ υ (C-O) |  | 1146 | 1101 | 122 |
| 58 | υ (C-O) + _+_ Ipb (C-H)Ar |  | 1162 | 1117 | 239 |
| 59 | (C-H)CH_2_ + υ (C-O) |  | 1182 | 1136 | 0.08 |
| 60 | Ipb(C-H)Ar |  | 1204 | 1157 | 0.69 |
| 61 | Ipb(C-H) Ar | 1103 | 1224 | 1176 | 10.64 |
| 62 | υ (C-O)+ δ(C-H)CH_2_ |  | 1263 | 1214 | 38 |
| 63 | δ(C-H)CH_2_ |  | 1296 | 1245 | 88 |
| 64 | υ (C-N) + Ipb(C-H)Ar |  | 1300 | 1249 | 16.5 |
| 65 | δ(C-H)CH_2_ |  | 1321 | 1269 | 32.7 |
| 66 | Ipb(C-H)Ar +δ(C-H)CH_2_ + υ (C-O) |  | 1335 | 1283 | 136 |
| 67 | Ipb(C-H)Ar+ δ(C-H)CH_2_ |  | 1345 | 1293 | 166 |
| 68 | . υ (C=N)+Ipb(C-H)+ δ (C-H)CH_2_ |  | 1357 | 1304 | 29.5 |
| 69 | υ (C-O)+ δ (C-H)CH_2_ |  | 1369 | 1316 | 84 |
| 70 | Ipb(C-H)CH_2_ + Ipb(C-H)Ar |  | 1397 | 1343 | 79 |
| 71 | Ipb (C-H)CH_2_ + Ipb(C-H)Ar |  | 1406 | 1351 | 61 |
| 72 | Ipb(C-H)CH_2_ + υ (C-O) |  | 1416 | 1361 | 20 |
| 73 | Ipb(C-H)CH_2_ |  | 1433 | 1377 | 40.4 |
| 74 | Ipb(C-H)CH_2_ |  | 1448 | 1392 | 35.3 |
| 75 | Ipb(C-H)CH_2_ + Ipb (O-H) |  | 1459 | 1402 | 18.16 |
| 76 | δ (C-H)CH_2_ |  | 1459 |  | 18.16 |
| 77 | δ (C-H)CH_2_ |  | 1470 | 1413 | 38.8 |
| 78 | υ (C=C) +(C-H)CH_2_ |  | 1479 | 1421 | 8.8 |
| 79 | δ(C-H)CH_3_ |  | 1481 | 1423 | 56.6 |
| 80 | υ (C=C) + δ(C-H)CH3+ τ (C-H)CH_2_ |  | 1491 | 1433 | 5.6 |
| 81 | τ (C-H)CH_2_ + υ(C=C) | 1430 | 1504 | 1445 | 111 |

**Table S6. Continue**.

| Mode | Compound 1 | | | | |
| --- | --- | --- | --- | --- | --- |
|  | Assignment | Exp | Cal | Scale | I IR |
| 82 | υ (C=C) + τ(C-H)CH_2_ |  | 1528 | 1468 | 149 |
| 83 | υ(C=N)+ υ(C=C) |  | 1543 | 1483 | 43 |
| 84 | υ(C=N)+ υ (C=C) |  | 1594 | 1564 | 294 |
| 85 | υ.(C=N) + υ(C=C) |  | 1624 | 1561 | 407 |
| 86 | υ.(C=N) + υ (C=C) |  | 1630 | 1566 | 42 |
| 87 | υ (C=C) | 1737 | 1642 | 1578 | 22 |
| 88 | υ (C=C)+ υ (C=C) |  |  |  |  |
| 89 | υ(C=O) |  | 1815 |  | 283 |
| 90 | υsym (C-H)CH_2_ |  | 3039 | 2917 | 15 |
| 91 | υsym (C-H)CH_2_ |  | 3090 | 2966 | 8.12 |
| 92 | υasym (C-H) CH_3_ |  | 3092 | 2968 | 23 |
| 93 | υsym (C-H) CH_3_ |  | 3116 | 2991 | 4.26 |
| 94 | υsym (C-H)CH_2_ |  | 3128 | 3003 | 8.23 |
| 95 | υsym (C-H)CH_3_ | 1431 | 3150 | 3024 | 2.77 |
| 96 | υsym (C-H)CH_3_ |  | 3166 | 3039 | 0.22 |
| 97 | υas(C-H) Aromatic |  | 3169 | 3042 | 15 |
| 98 | υas(C-H) pyrimidine |  | 3176 | 3049 | 17 |
| 99 | υas(C-H) Aromatic |  | 3190 | 3062 | 24 |
| 100 | υs(C-H) Aromatic |  | 3226 | 3097 | 0.748 |
| 101 | υs(C-H) Aromatic |  | 3230 | 3101 | 7.5 |
| 102 | υ O-H | 3402 | 3752 | 3602 | 71.7 |
|  |  |  |  |  |  |

σ: Scissoring t: Torision τ: Twisting ω: Waging υs: Symmetric stretching

υas Asymmetric stretching δ: Rocking Ipb: In the plane bending Asb: Asymmetric bending

Opb: Out of the plane bending υ: Streching Sb: Symmetric bending

**Table S7.** Observed and calculated vibrational frequency of compound **(2**) at B3LYP method with 6-311+G(d,p).

| Mode | Compound 2 | | | | | | | | | |
| --- | --- | --- | --- | --- | --- | --- | --- | --- | --- | --- |
|  | Assignment | | Exp | | Cal | | Scale | | I IR | |
| 20 | γ (C- C) | |  | | 379 | | 364 | | 8.8 | |
| 21 | γ (C-C) | |  | | 416 | | 400 | | 0.07 | |
| 22 | γ (C- C-C) | |  | | 429 | | 412 | | 3.77 | |
| 23 | W (O-C-N-C) + Out of the plane + bending CO+ T( NH2) | |  | | 481 | | 462 | | 8 | |
| 24 | γ (C- C-C) | |  | | 510 | | 490 | | 2.89 | |
| 25 | W (NH2) + bending CO | |  | | 523 | | 503 | | 19.22 | |
| 26 | γ (C- C-C) | |  | | 535 | | 514 | | 10.47 | |
| 27 | δ (C- C-N) | |  | | 569 | | 547 | | 12 | |
| 28 | δ (C- C-N)+ W (NH2) | |  | | 598 | | 575 | | 7.78 | |
| 29 | γ (C- C-C) + W (NH2) | |  | | 607 | | 583 | | 6.5 | |
| 30 | γ (C- C-C) | |  | | 632 | | 607 | | 0.44 | |
| 31 | γ (C- C-C) + δ (C-C-N) | |  | | 650 | | 625 | | 1.14 | |
| 32 | In the plane (C- C-C)ph + δ (C-C-N) | |  | | 656 | | 630 | | 1.15 | |
| 33 | In the plane (C- C-C) + δ NH2 | |  | | 684 | | 657 | | 1.36 | |
| 34 | In the plane (C- C-C) + δ (C-C-N) | |  | | 690 | | 663 | | 49 | |
| 35 | In the plane (C- C-C) **+** δ (C-C-N) | |  | | 696 | | 669 | | 6.14 | |
| 36 | In the plane (C- C-C)ph | |  | | 705 | | 678 | | 22.27 | |
| 37 | In the plane (C- C-C)ph **+** δ (C-C-N) | |  | | 729 | | 701 | | 2.08 | |
| 38 | In the plane (C- C-C)ph **+** δ (C-C-N )+opb(C-H)Ar+ δ(C-H)CH_2_ | |  | | 769 | | 739 | | 52 | |
| 39 | opb(C-H)Ar + In the plane (C- C-C)ph | |  | | 793 | | 762 | | 2.99 | |
| 40 | υ(C-O) +υ (C=N)+ In the plane (C- C-C)ph | |  | | 803 | | 772 | | 15.49 | |
| 41 | opb(C-H)Ar + In the plane (C- C-C)ph + δ(C-H)CH_3_ | |  | | 808 | | 776 | | 7 | |
| 42 | opb(C-H)Ar | |  | | 855 | | 822 | | 0.12 | |
| 43 | υ (C=N)+ C-O + δ(C-H)CH_2_ | |  | | 860 | | 826 | | 7.34 | |
| 44 | In the plane (C- C-C)ph +opb(C-H)Ar+ δ (C-C-N) | |  | | 933 | | 897 | | 31 | |
| 45 | opb(C-H) Ar | |  | | 934 | | 898 | | 6.52 | |
| 46 | opb(C-H) Py | |  | | 977 | | 939 | | 3.7 | |
| 47 | δ(C-H)CH_2_ | |  | | 991 | | 952 | | 0.36 | |
| 48 | δ(C-H)CH_2_ | |  | | 998 | | 959 | | 17 | |
| 49 | δ(C-H)CH_2_ | |  | | 1000 | | 961 | | 0.49 | |
| 50 | υ (C-H) CH_3_+opb(C-H)Ar+δ(C-H)CH_2_+ δ NH_2_ | |  | | 1005 | | 966 | | 56 | |
| 51 | (C-H) Pyr | |  | | 1016 | | 976 | | 17 | |
| 52 | υ (C-C) | |  | | 1034 | | 994 | | 22 | |
| 53 | δ(C-H)CH_2_+ Ipb (C-H)Ar+ N-H | |  | | 1049 | | 1008 | | 7.4 | |
| 54 | δ(C-H)CH_2_+ Ipb (C-H)Ar+ υas N-H | |  | | 1055 | | 1014 | | 6.78 | |
| 55 | δ (C-H) CH3 | |  | | 1060 | | 1019 | | 1.22 | |
| 56 | Ipb (C-H)Ar+ N-N | |  | | 1092 | | 1049 | | 26 | |
| 57 | υ (C-C)+ Ipb (C-H)Ar+ υ (C-O) | |  | | 1093 | | 1050 | | 7.16 | |
| 58 | υ (C-N) +δ(C-H)CH_2_+ δ N-H | |  | | 1117 | | 1073 | | 11.26 | |
| 59 | υN-N+υ (C-O)+ Ipb(C-H)Ar | |  | | 1124 | | 1080 | | 146 | |
| 60 | υN-N+υ (C-O)+ Ipb(C-H)Ar | |  | | 1146 | | 1101 | | 94 | |
| 61 | Ipb(C-H)Ar | |  | | 1182 | | 1136 | | 0.02 | |
| 62 | Ipb(C-H)Ar | |  | | 1205 | | 1158 | | 0.8 | |
| 63 | δ(C-H)CH_2_ | | 1103 | | 1229 | | 1181 | | 20 | |
| 64 | δ(C-H)CH_2_ | |  | | 1261 | | 1212 | | 33 | |
| 65 | δ(C-H)CH_2_+ υ(C=N) | |  | | 1293 | | 1243 | | 47 | |
| 66 | υ (C-O)+ Ipb (C-H)Ar + (C-N) | |  | | 1296 | | 1245 | | 83 | |
| 67 | Ipb (C-H)Ar+ δ(C-H)CH_2_+ υ(C=N) | |  | | 1321 | | 1269 | | 66 | |
| 68 | Ipb(C-H)Ar + C-H)CH_2_ | |  | | 1334 | | 1282 | | 181 | |
| 69 | Ipb (C-H)Ar+ δ(C-H)CH_2_+ υ(C=N) | |  | | 1344 | | 1292 | | 152 | |
| 70 | Ipb(C-H)Ar | |  | | 1357 | | 1304 | | 30 | |
| 71 | Ipb (C-H)Ar+ T(C-H)CH_2_+ υ(C=N) | |  | | 1369 | | 1316 | | 81 | |
| 72 |  | |  | |  | |  | |  | |
| 73 | υ (C-N + δ(C-H)CH_2_ | |  | | 1405 | | 1350 | | 67 | |
| 74 | Ipb(C-H)+ δ(C-H)CH_2_ | |  | | 1414 | | 1359 | | 5.8 | |
| 75 | δ(C-H)CH_2_ | |  | | 1432 | | 1376 | | 48 | |
| 76 | | υ (C=C)+υ(C=N)+)+ δ(C-H)CH_2_ | |  | | 1448 | | 1392 | | 37 |
| 77 | | υ (C-O) + υ (C=N)+ (C-H)CH_2_ + υ (C-N) | |  | | 1467 | | 1410 | | 14.05 |
| 78 | | υ (C=N)+ υ (C-O)+ δ (C-H)CH_2_ | |  | | 1470 | | 1413 | | 43 |
| 79 | | T(C-H)CH_2_ | |  | | 1479 | | 1421 | | 11.85 |

**Table S7.** Continue.

| Mode | Compound 2 | | | | |
| --- | --- | --- | --- | --- | --- |
|  | Assignment | Exp | Cal | Scale | I IR |
| 80 | Ipb(C-H)CH_3_ | 1262 | 1481 | 1423 | 51 |
| 81 | υ (C=N)+ υ (C-H) CH_3_+ υ( C=C) |  | 1491 | 1433 | 7.4 |
| 82 | Ipb(C-H)Ar+υ (C=N)+ υ (C=C) |  | 1505 | 1446 | 111.44 |
| 83 | Ipb(C-H)CH_2_+ υ.(C=N) +υ(C=C) |  | 1528 | 1468 | 152 |
| 84 Ipb(C-H)CH_2_ |  |  | 1544 | 1484 | 43 |
| 85 | υ.(C=N) +υ(C=C) |  | 1594 | 1532 | 285 |
| 86 | .υ (C=N) +υ(C=C) + Ipb(C-H)CH_2_ |  | 1620 | 1557 | 183 |
| 87 | υ(C=C) + υ (C=N)+ δ NH2 |  | 1624 | 1561 | 350 |
| 88 | υ.(C=N) +υ(C=C) |  | 1630 | 1566 | 32 |
| 89 | υ.(C=N) +υ(C=C) |  | 1643 | 1579 | 22.24 |
| 90 | C=O (amide ) | 1673 | 1763 | 1694 | 340 |
| 91 | υsym (C-H) CH_3_ |  | 3039 | 2978 | 15 |
| 92 | υs(C-H) CH_2_ |  | 3040 | 2918 | 6.8 |
| 93 | υasym (C-H)CH_2_ CH_2_ |  | 3089 | 2965 | 6.3 |
| 94 | υasym (C-H)CH3 | 1431 | 3091 | 2967 | 8.2 |
| 95 | υasym (C-H)CH_2_ |  | 3098 | 2974 | 28 |
| 96 | υasym (C-H)CH_2_ | 1447 | 3128 | 3003 | 8.21 |
| 97 | υasym (C-H)CH_2_ |  | 3152 | 3026 | 2.58 |
| 98 | υas(C-H)phen |  | 3166 | 3039 | 0.27 |
| 99 | (C=N)+υ s(C-H)pyr |  | 3168 | 3041 | 16.38 |
| 100 | υs(C-H) Aromatic |  | 3176 | 3049 | 17.18 |
| 101 | υs(C-H) Aromatic |  | 3190 | 3062 | 23.6 |
| 102 | υas(C-H) Aromatic |  | 3226 | 3097 | 0.8 |
| 103 | υas(C-H) Aromatic |  | 3231 | 3102 | 8.56 |
| 104 | υs NH_2_ | 3402 | 3589 | 3445 | 38 |
| 105 | υasNH_2_ | 3368 | 3724 | 3575 | 39 |

**Table S8.** Observed and calculated vibrational frequency of compound **(3**) at B3LYP method with 6-311+G(d,p).

| Mode | Compound 3 | | | | |
| --- | --- | --- | --- | --- | --- |
|  | Assignment | Exp | Cal | Scale | I IR |
| 20 | γ (C- C) |  | 322 | 309 | 3.2 |
| 21 | γ (C-C) |  | 354 | 340 | 10.35 |
| 22 | γ (C- C-C) |  | 361 | 347 | 10.03 |
| 23 | W (O-C-N-C) + Out of the plane |  | 369 | 355 | 0.158 |
| 24 | Out of the plane bending CO + |  | 403 | 387 | 15.72 |
| 25 | γ (C- C) |  | 415 | 399 | 0.12 |
| 26 | γ (C- C-C) |  | 448 | 431 | 6.86 |
| 27 | γ (C- C-C) |  | 510 | 490 | 2.45 |
| 28 | γ (C- C-C)+ deformation γ (C- O-C) bending CO |  | 517 | 497 | 11.58 |
| 29 | γ (C- C-C) |  | 536 | 515 | 5.9 |
| 30 | δ (C- C-N) |  | 568 | 546 | 11.09 |
| 31 | Out of the plane bending CO + deformation (C-O-C) |  | 589 | 566 | 4.14 |
| 32 | W (O-C-N-C) + Out of the plane bending CO+ δ (C-C-N) |  | 605 | 581 | 6.2 |
| 33 | In the plane (C- C-C)ph |  | 632 | 607 | 0.36 |
| 34 | In the plane (C- C-C)ph + δ (C-C-N) |  | 650 | 625 | 1.94 |
| 35 | In the plane (C- C-C)ph **+** δ (C-C-N) |  | 655 | 629 | 0.56 |
| 36 | δ(C-H)CH_2_ |  | 668 | 642 | 0.10 |
| 37 | In the plane (C- C-C)ph **+** δ (C-C-N) |  | 691 | 664 | 50 |
| 38 | In the plane (C- C-C)ph **+** δ (C-C-N |  | 694 | 667 | 5 |
| 39 | opb(C-H)Ar |  | 705 | 678 | 23.08 |
| 40 | δ(C-H)CH_2_  + opb(C-H)Ar |  | 732 | 703 | 2.3 |
| 41 | opb(C-H)Ar |  | 769 | 739 | 52 |
| 42 | Copb(C-H)Ar+δ(C-H)CH_2_ |  | 795 | 764 | 4.06 |
| 43 | υ (C=N) |  | 801 | 770 | 18.4 |
| 44 | υ (C=N) +opb(C-H)Ar+ δ (C-C-N) |  | 808 | 776 | 7.45 |
| 45 | opb(C-H) Ar |  | 855 | 822 | 0.03 |
| 46 | δ(C-H)CH_2_ |  | 860 | 826 | 13.6 |
| 47 | δ(C-H)CH_2_ |  | 865 | 831 | 2.5 |
| 48 | δ(C-H)CH_2_ |  | 891 | 856 | 7.06 |
| 49 | (opb(C-H)Ar |  | 935 | 899 | 6 |
| 50 | υ (C-H)CH_3_+opb(C-H)Ar+δ(C-H)CH_2_ |  | 936 | 899 | 32 |
| 51 | υ (C-H) Pyr |  | 977 | 939 | 3.6 |
| 52 | Ipb (C-H)Ar |  | 991 | 952 | 0.12 |
| 53 | δ(C-H)CH_2_ |  | 998 | 959 | 10.88 |
| 54 | Ipb (C-H)Ar |  | 1000 | 961 | 0.6 |
| 55 | Ipb (C-H)Ar+ υ (C-O) |  | 1013 | 973 | 66 |
| 56 | Ipb (C-H)Ar+ υ (C-O) |  | 1016 | 976 | 29 |
| 57 | δ(C-H)CH_2_+ Ipb (C-H)Ar+ υ (C-O |  | 1032 | 992 | 36 |
| 58 | Ipb C-H) CH3+Ipb(C-H)Ar+δ(C-H)CH_2_ |  | 1035 | 995 | 28 |
| 59 | Ipb (C-H)Ar |  | 1051 | 1010 | 0.8 |
| 60 | Ipb (C-H) CH3 |  | 1060 | 1019 | 0.96 |
| 61 | δ(C-H)CH_2_+ Ipb(C-H)Ar | 1103 | 1091 | 1048 | 42 |
| 62 | υ (C-O))+ δ(C-H)CH_2_ |  | 1093 | 1050 | 0.97 |
| 63 | υ (N-N)+ Ipb (C-H)+ δ(C-H)CH_2_ |  | 1094 | 1051 | 11.39 |
| 64 | υ (C=N) + Ipb (C-H)Ar+ υ (C-H)CH_2_+ υ (C-H) CH_2_CH_3_ |  | 1115 | 1072 | 33 |
| 65 | Ipb (C-H)Ar+ υ (C-O) |  | 1124 | 1080 | 162 |
| 66 | υ (C-O)+ Ipb (C-H)Ar |  | 1146 | 1101 | 106 |
| 67 | (Ipb (C-H)Ar+ |  | 1182 | 1136 | 2.3 |
| 68 | Ipb(C-H)Ar + υ(C-H)CH_2_ |  | 1185 | 1139 | 138 |
| 69 | Ipb(C-H)Ar +υ )C-H)CH_2_ + υ (C-O) |  | 1203 | 1156 | 145 |
| 70 | Ipb(C-H)Ar |  | 1206 | 1159 | 10 |
| 71 | υ (C-O)+ + δ(C-H)CH_2_ |  | 1239 | 1191 | 128 |
| 72 | υ (C-N + Ipb(C-H)Ar |  | 1264 | 1215 | 42 |
| 73 | υ (C-N + Ipb(C-H)Ar |  | 1295 | 1244 | 86.26 |
| 74 | Ipb(C-H)+ δ(C-H)CH_2_ + υ(C=N) |  | 1312 | 1261 | 38 |
| 75 | Ipb(C-H)+ δ(C-H)CH_2_ |  | 1333 | 1281 | 25 |
| 76 | υ (C-N) + (Ipb(C-H)Ar+ δ(C-H)CH_2_ |  | 1334 | 1282 | 165 |
| 77 | (C=N)+ (C-O)+Ipb(C-H)+ δ(C-H)CH_2_ |  | 1344 | 1292 | 149 |
| 78 | υ (C=N)+Ipb(C-H)CH_2_ + Ipb(C-H)Ar |  | 1357 | 1304 | 39 |

**Table S8. Continue**

| Mode | Compound 3 | | | | |
| --- | --- | --- | --- | --- | --- |
|  | Assignment | Exp | Cal | Scale | I IR |
| 79 | Ipb(C-H)Ar | 1262 | 1370 | 1317 | 86 |
| 80 | δ (C-H)CH_2_ |  | 1384 | 1330 | 42 |
| 81 | Ipb(C-H)CH_2_CH3 |  | 1397 | 1343 | 87 |
| 82 | Ipb(C-H)CH_2_+ Ipb(C-H)Ar |  | 1404 | 1349 | 33.6 |
| 83 Ipb(C-H)CH_2_ |  |  | 1415 | 1360 | 8.44 |
| 84 | υ(C=N) + Ipb(C-H)CH_2_ |  | 1422 | 1367 | 7.21 |
| 85 | υ(C=N) + Ipb(C-H)CH_2_ |  | 1433 | 1377 | 45 |
| 86 | υ (C=C )+ υ (C=N) |  | 1447 | 1391 | 38 |
| 87 | T (C-H)CH_2_ |  | 1461 | 1404 | 17 |
| 88 | υ (C=C) + υ (C=N)+ T (C-H)CH_2_ |  | 1470 | 1413 | 40 |
| 89 | υsym (C-H) CH_3_ |  | 1478 | 1420 | 11 |
| 90 | υ(C=C) |  | 1481 | 1453 | 9.48 |
| 91 | υasym (C-H) CH_3_ |  | 1488 | 1430 | 54 |
| 92 | υ(C=C ) |  | 1491 | 1433 | 6.05 |
| 93 | υsym (C-H) CH_3_ |  | 1493 | 1435 | 5.09 |
| 94 | υ(C=C) + υasym (C-H)CH_2_ | 1431 | 1505 | 1446 | 108 |
| 95 | υasym (C-H)CH3 |  | 1507 | 1448 | 13 |
| 96 | υ(C=C )+ υ (C=N)+υas(C-H)phen |  | 1527 | 1467 | 161 |
| 97 | υ(C=C )+ υas(C-H)phen |  | 1544 | 1484 | 42 |
| 98 | υ(C=C )+υ (C=N)+υas(C-H)phen |  | 1594 | 1532 | 294 |
| 99 | υ(C=C + υ (C=N)+υ s(C-H)phen |  | 1624 | 1561 | 420 |
| 100 | υs(C-H) Aromatic + υ( C=C ) |  | 1630 | 1566 | 43 |
| 101 | υs(C-H) Aromatic + υ(C=C) |  | 1643 | 1579 | 22 |
| 102 | υ C=O Ester | 1741 | 1786 | 1716 | 212 |
| 103 | υs(C-H) CH_2_ |  | 3034 | 2913 | 6 |
| 104 | υs(C-H) (CH_2_CH_3_) |  | 3035 | 2914 | 19.5 |
| 105 | υs(C-H) CH3 |  | 3039 | 2917 | 15.4 |
| 106 | υs(C-H) CH_2_ (CH_2_CH_3_) |  | 3074 | 2951 | 16.5 |
| 107 | υas(C-H) CH_3_ |  | 3090 | 2966 | 8.4 |
| 108 | υas(C-H) CH_2_ |  | 3093 | 2969 | 23 |
| 109 | υas(C-H) CH_2_CH_3_ |  | 3099 | 2975 | 25 |
| 110 | υas(C-H) CH_2_CH_3_ |  | 3108 | 2984 | 8 |
| 111 | υas(C-H) CH_2_ |  | 3119 | 2994 | 4.7 |
| 112 | υas(C-H) CH_3_ |  | 3128 | 3003 | 8.4 |
| 113 | υas(C-H) CH_2_CH_3_ |  | 3132 | 3007 | 20 |
| 114 | υas(C-H) CH_2_ |  | 3151 | 3025 | 3 |
| 115 | υs(C-H) Aromatic |  | 3165 | 3038 | 0.2 |
| 116 | υs(C-H) pyrimidine |  | 3169 | 3042 | 16.17 |
| 117 | υas(C-H) Aromatic |  | 3175 | 3048 | 17.6 |
| 118 | υs(C-H) Aromatic |  | 3189 | 3061 | 24 |
| 119 | υs(C-H) Aromatic |  | 3227 | 3098 | 1.05 |
| 120 | υas(C-H) Aromatic |  | 3231 | 3102 | 10 |

**
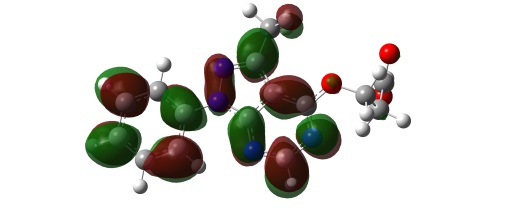
Supplementary Figures.**

**(LUMO+6)**

**-0.409 eV**


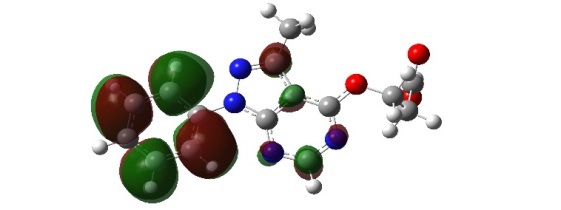


**-0.517 eV**

**(LUMO+3)**

**
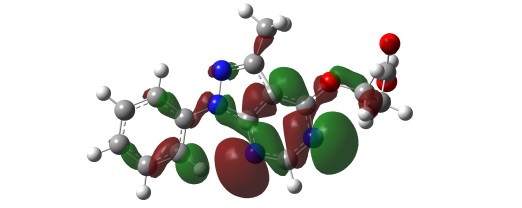
**
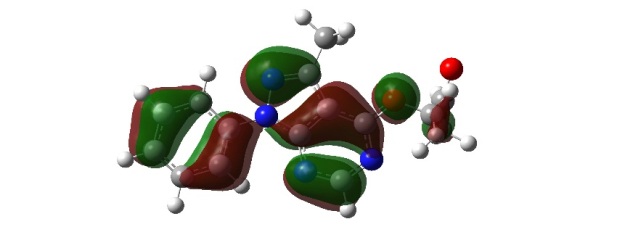

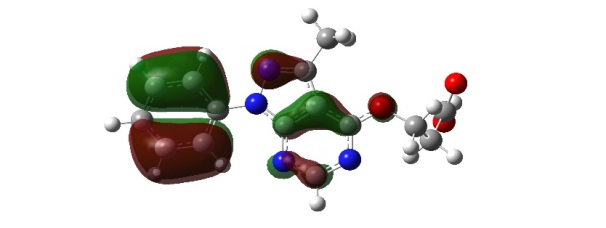

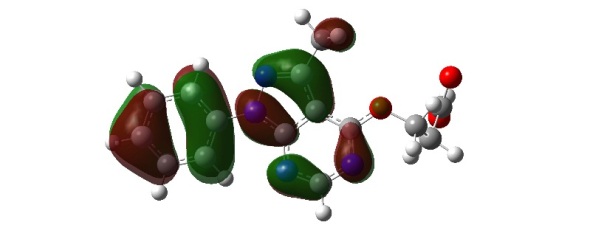

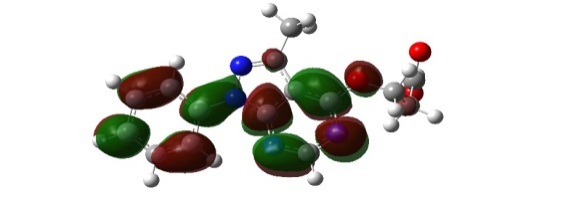

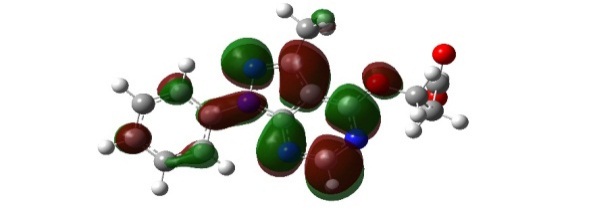

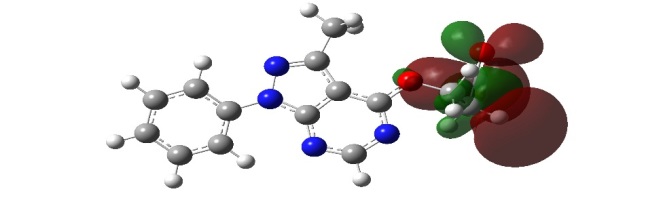


**[Fig.S1.** Molecular orbital diagram of the titled compound 1 with main contribution to electronic excitation obtained from TD-DFT calculations.

**(HOMO-3)**

**-7.697 eV**

**(LUMO)**

**(HOMO)**

**-6.261 eV**

**-1.611 eV**

**-7.207 eV**

**(HOMO-2)**

**(LUMO+1)**

**(HOMO-1)**

**(LUMO+2)**

**-7.081 eV**

**-0.997 eV**

**-0.660 eV**


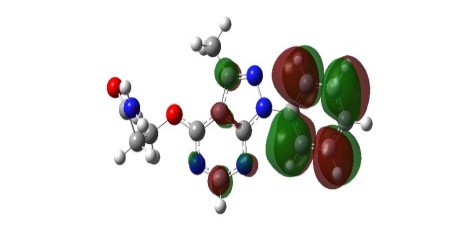
**
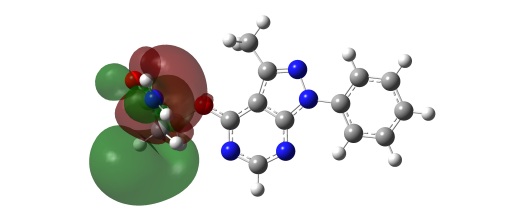
**

**(LUMO+6)**

**-0.245 eV**

**-0.524 eV**

**(LUMO+3)**


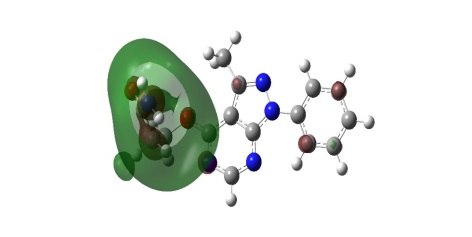


**-1.615 eV**

**(LUMO+1)**

**(LUMO+2)**

**-1.00 eV**

**-0.624 eV**


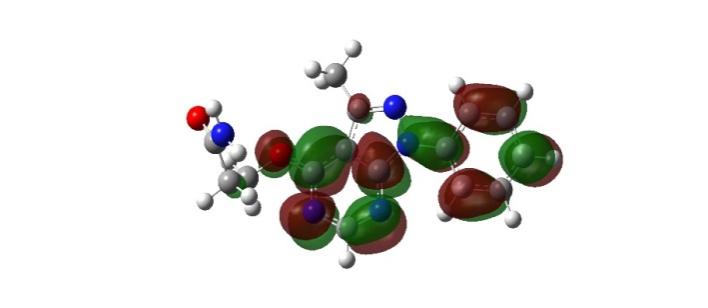


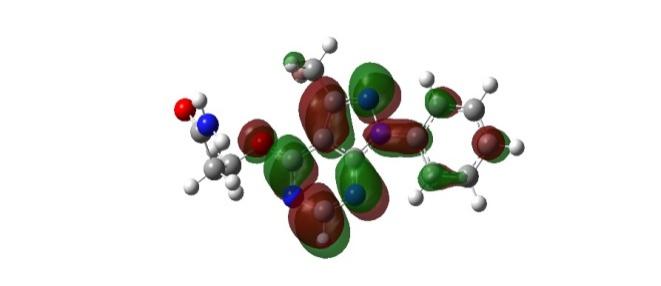


**(LUMO)**


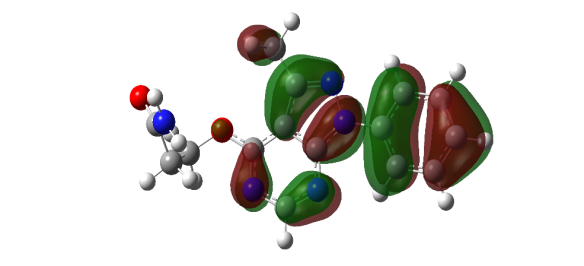


**-6.262 eV**

**(HOMO)**


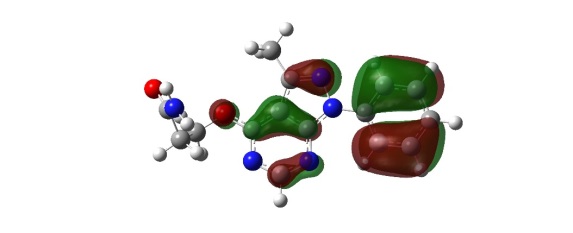


**-7.088 eV**

**(HOMO-1)**


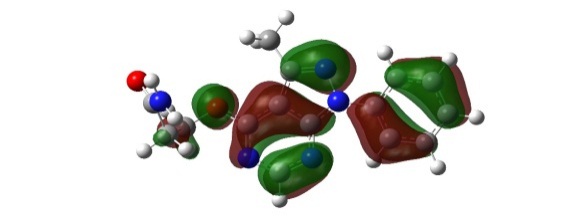


**(HOMO-2)**

**-7.196 eV**


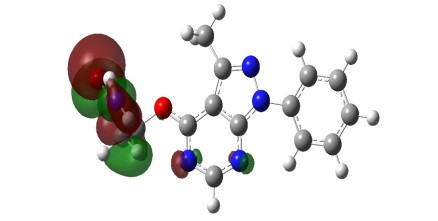


**(HOMO-3)**

**-7.436 eV**

**
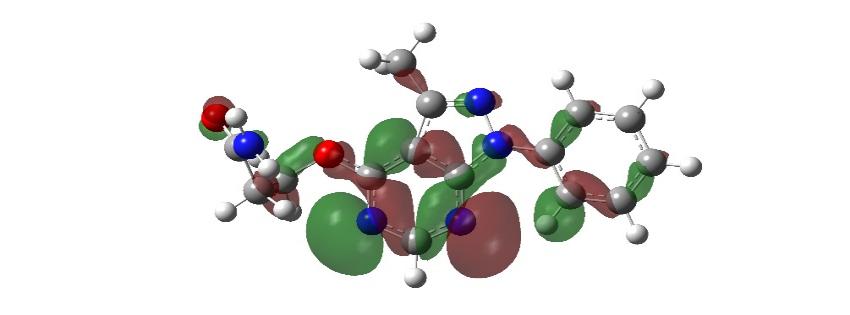
**

**(HOMO-4)**

**-7.70 eV**

**Fig. S2.** Molecular orbital diagram of the compound 2 with main contribution to electronic excitation obtained from TD-DFT calculations.**.**

**
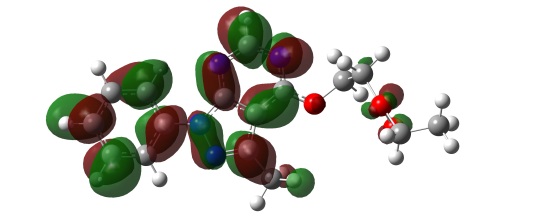
**

**(LUMO+6)**

**-0.179 eV**


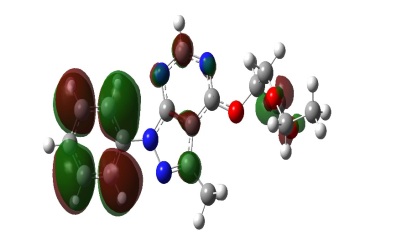


**-0.480 eV**

**(LUMO+3)**


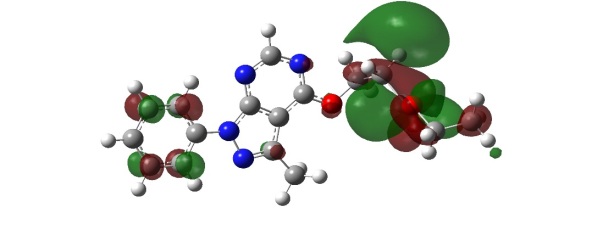

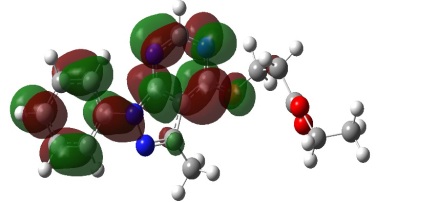

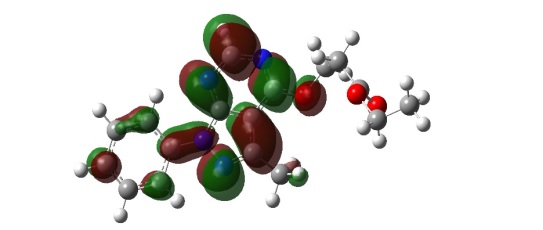

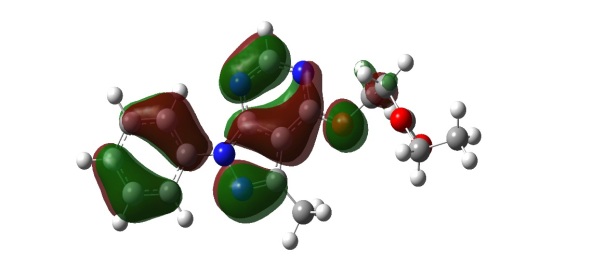

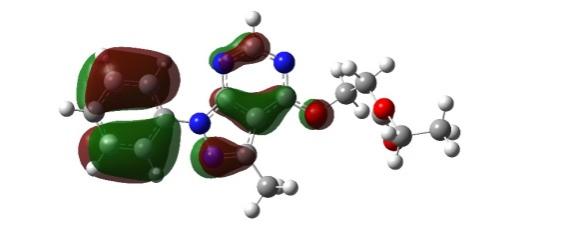

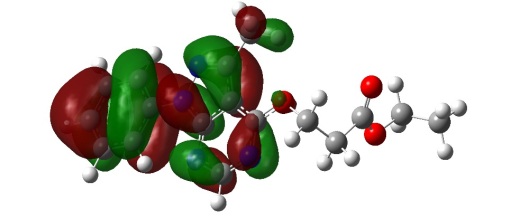


**(HOMO-3)**

**-7.636 eV**

**-6.214 eV**

**(LUMO)**

**-1.566 eV**

**-7.156 eV**

**(HOMO-2)**

**(LUMO+1)**

**(HOMO-1)**

**(LUMO+2)**

**-7.043 eV**

**-0.949 eV**

**-0.527 eV**

**(HOMO)**


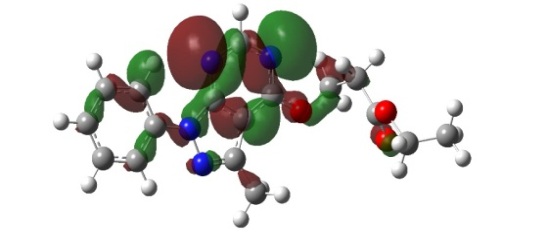


**[**

**[Fig.S3.** Molecular orbital diagram of the compound 3 with main contribution to electronic excitation obtained from TD-DFT calculations.
